# Preexisting symptoms increase the risk of developing long COVID during the SARS‐CoV‐2 pandemic

**DOI:** 10.1111/joim.20102

**Published:** 2025-06-04

**Authors:** Vincent Lak, Helen Sjöland, Martin Adiels, Christina E. Lundberg, Josefina Robertson, Maria Åberg, Christian Alex, Martin Lindgren, Annika Rosengren

**Affiliations:** ^1^ Department of Molecular and Clinical Medicine, Institute of Medicine Sahlgrenska Academy, University of Gothenburg Gothenburg Sweden; ^2^ Department of Medicine Geriatrics and Emergency Medicine Sahlgrenska University Hospital Östra Hospital, Region Västra Götaland Gothenburg Sweden; ^3^ School of Public Health and Community Medicine, Institute of Medicine, University of Gothenburg Gothenburg Sweden; ^4^ Department of Food and Nutrition and Sport Science, Faculty of Education University of Gothenburg Gothenburg Sweden; ^5^ General Practice/Family Medicine, School of Public Health and Community Medicine, Institute of Medicine, Sahlgrenska Academy, University of Gothenburg Gothenburg Sweden; ^6^ Department of Infectious Diseases, Institute of Biomedicine Sahlgrenska Academy, University of Gothenburg Gothenburg Sweden; ^7^ Department of Infectious Diseases Sahlgrenska University Hospital, Region Västra Götaland Gothenburg Sweden; ^8^ Region Västra Götaland, Regionhälsan Gothenburg Sweden

**Keywords:** clinical coding, long COVID, post‐acute sequelae of COVID‐19 (PASC), post‐COVID‐19 syndrome, primary health care

## Abstract

**Background:**

Long COVID is defined as otherwise unexplained symptoms following a SARS‐CoV‐2 infection.

**Objective:**

To examine the prevalence of preexisting symptoms compatible with long COVID in individuals with a diagnosis of long COVID.

**Methods:**

This retrospective, observational study included the adult population (aged 18 years and older) in Region Västra Götaland, with at least one recorded healthcare contact between January 1, 2020, and November 30, 2023, from a regional database comprising all levels of healthcare contacts. Data on long COVID, relevant symptoms before and after the pandemic started (2016–2023), and SARS‐CoV‐2 infection status were extracted using the International Classification of Diseases version 10 (ICD‐10) codes. Individuals who had been hospitalized due to a SARS‐CoV‐2 infection were considered separately.

**Results:**

Out of 1,415,885 individuals, 9202 (0.6%) had been diagnosed with long COVID. Among the non‐hospitalized individuals, the record of at least one of the relevant symptoms was more common in those with long COVID compared to those without it (57.6% vs. 36.3% for men and 71.6% vs. 50.4% for women), already before January 1, 2020. Among individuals with any relevant symptom, the odds ratios (ORs) of having long COVID were OR = 2.28 (95% confidence interval [CI] = 2.10–2.48) for men and OR = 2.32 (95% CI = 2.18–2.48 for women) after adjusting for age group, obesity, asthma, and anxiety, compared with individuals without any relevant symptom.

**Conclusions:**

Individuals diagnosed with long COVID had more healthcare contacts for relevant symptoms even before the pandemic compared to individuals without long COVID.

## Graphical abstract

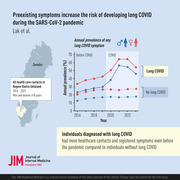

## Introduction

Long COVID is defined by the World Health Organization (WHO) as the continuation or development of new symptoms 3 months after the initial SARS‐CoV‐2 infection, with symptoms lasting for at least 2 months and with no other explanation, and where symptoms generally have an impact on everyday functioning [1]. More than 200 symptoms in ten organ systems have been described as possibly relevant to long COVID [2]. In the WHO Delphi consensus process of a clinical case definition of long COVID, 13 out of 24 symptoms (shortness of breath, cough, tachycardia, fatigue, post‐exertional malaise, cognitive dysfunction, impaired memory, sleep disorders, loss of smell/taste, headache, chest pain, muscle spasms, and joint pain) were identified as important by more than 50% of the participants [1]. The International Classification of Diseases version 10 (ICD‐10) code U09.9 for long COVID was introduced in Sweden by mid‐October 2020 [3].

Even so, more than 3 years after the publication of the WHO definition of the post‐COVID‐19 condition (the WHO official name for long COVID), there is no universal consensus on the name or exact definition of the persisting sequelae of SARS‐CoV‐2 infections, known as ongoing symptomatic COVID‐19 or post‐COVID‐19 syndrome in the United Kingdom and as long COVID in the United States [4]. For brevity, we will use the latter term in the following. Moreover, studies differ in size, setting, selection process, symptom selection, and recording. Comprehensive reviews and meta‐analyses report vastly different estimates on the overall prevalence of long COVID, ranging between 6% and 57%, with higher prevalence in patients hospitalized compared with non‐hospitalized for SARS‐CoV‐2 [5, 6].

Unlike acute SARS‐CoV‐2 infections, which can be established using laboratory methods, there is no objective measurement underpinning long COVID. Apart from the criterion of a past infection, the diagnosis rests on the presence of symptoms that are common in the general population. Whether individuals presenting with long COVID demonstrate greater susceptibility to symptoms compatible with long COVID than other people prior to the pandemic has not been established. Previous studies have predicted substantial increases in healthcare costs and utilization from patients with long COVID [7, 8]. However, few studies have estimated the prevalence and healthcare utilization of long COVID in adults using data from an entire region over an extended period.

The purpose of this study was to estimate, first, the proportion of the population registered with ICD codes for any of the relevant symptoms before and during the pandemic; second, the long COVID prevalence in the Region Västra Götaland, Sweden; and third, healthcare contacts of patients registered with a diagnosis of long COVID.

## Methods

### Study design

This was a retrospective, observational study based on a regional database of registered and completed healthcare contacts to physicians and other healthcare professionals.

### Data source

We obtained data from the regional database of healthcare contacts of the Region Västra Götaland (VEGA), a comprehensive regional database covering all hospital admissions, outpatient specialist care, and primary care contacts in the Region Västra Götaland, Sweden, to any healthcare professional [9]. The database also contains information on all hospital‐based healthcare contacts of residents of the Region Västra Götaland outside of the region. The adult population in the Region Västra Götaland is approximately 1.4 million.

### Inclusion

We included all individuals in the Region Västra Götaland aged 18 years or older with at least one registered healthcare contact from January 1, 2020, to November 30, 2023.

### Variables assessed

Long COVID diagnosis was defined by the ICD‐10 code U09.9. Acute SARS‐CoV‐2 infection was defined by the ICD‐10 codes U07.1 and U07.2. We use the shorthand “the relevant symptoms” to refer to the 13 symptoms that were identified as important to the clinical case definition of the post‐COVID‐19 condition by more than 50% of the participants of the WHO Delphi consensus process. We categorized these into 10 symptom entities and 3 symptom groups: cardiopulmonary (dyspnea, cough, palpitations), from the central nervous system (CNS) (fatigue, cognitive dysfunction, sleep disorder, loss of smell/taste), and pain (headache, chest pain, muscle/joint pain). Symptoms were identified by ICD‐10 codes registered during 2016–2023 (Table S1).

Age was defined as the year of birth subtracted from 2020 and grouped into 18–39, 40–59, 60–79, and 80+. Baseline comorbidities, registered from January 1, 2015, to December 31, 2019, were retrieved by ICD‐10 codes (Table S2). We separated the study population into those who had been hospitalized with SARS‐CoV‐2, defined by an inpatient hospital visit with a diagnosis of SARS‐CoV‐2, and those who had not been hospitalized, with the latter forming the main focus for the analyses and referred to as the non‐hospitalized population. We defined any use of either mechanical ventilation by endotracheal intubation (identified by the Swedish procedure coding system [KVÅ] codes DG017 or DG018) or heated humidified high‐flow nasal oxygen therapy (code DG028) as a marker of severe infection.

Group comparisons were made between the individuals with and without a long COVID diagnosis. We defined 2016–2019 as the pre‐pandemic and 2020–2023 as the pandemic period. Prevalence of symptoms in the long COVID group was compared with the no long COVID group in the pre‐pandemic and pandemic periods as well as the annual prevalence, which was defined as the number of individuals with at least one healthcare contact for one of the relevant symptoms during any particular year divided by the total number of individuals in that group. Results for men and women are presented separately.

Symptom prevalence was subjected to a sensitivity analysis where we classified the individuals with long COVID diagnosis dated 90 days or later after a SARS‐CoV‐2 infection diagnostic code, consistent with the WHO definition, as the “long COVID ≥90 days after” group; and the individuals with a previous SARS‐CoV‐2 infection but without fulfilling the criterion of a long COVID diagnosis registered at least 90 days after the infection as the “no long COVID ≥90 days after” group.

### Statistical analysis

Group comparison *p*‐values are derived from the *chi*‐squared test for categorical variables and the independent *t*‐test for continuous variables when comparing mean values. The association between pre‐pandemic symptoms and long COVID in the non‐hospitalized population was analyzed using logistic regression. First, an unadjusted, univariate model; second the fully adjusted multivariate model, which included age as well as a selection of the baseline comorbidities. To avoid variables with very low frequencies, we included symptoms and baseline comorbidities that had a count of 100 individuals or more in the long COVID group and a statistically significant difference in prevalence compared with the no long COVID group, that is, with a *p*‐value <0.05, in both men and women. A correlation matrix with Pearson correlation coefficients was then used to identify baseline comorbidities that had at least a moderate correlation (*r* ≥ 0.4). To avoid issues with multicollinearity, when we had correlated baseline comorbidities, we kept only the one with the highest prevalence in the long COVID group. Outcomes from logistic models were presented as odds ratios (ORs) and 95% confidence intervals (95% CIs). Logistic regression was used to adjust for age in group comparisons on prevalence differences in baseline comorbidities. Negative binomial regression was used to adjust for confounders in group comparisons on the number of healthcare contacts. Statistical calculations were performed using R version 4.3.0 software (http://www.R‐project.org).

### Ethics statement

The study conforms to the principles outlined by the Declaration of Helsinki and was approved by the Swedish Ethical Review Authority (Dnr: 2021‐01692, 2023‐05002‐02).

## Results

**Fig. 1:**
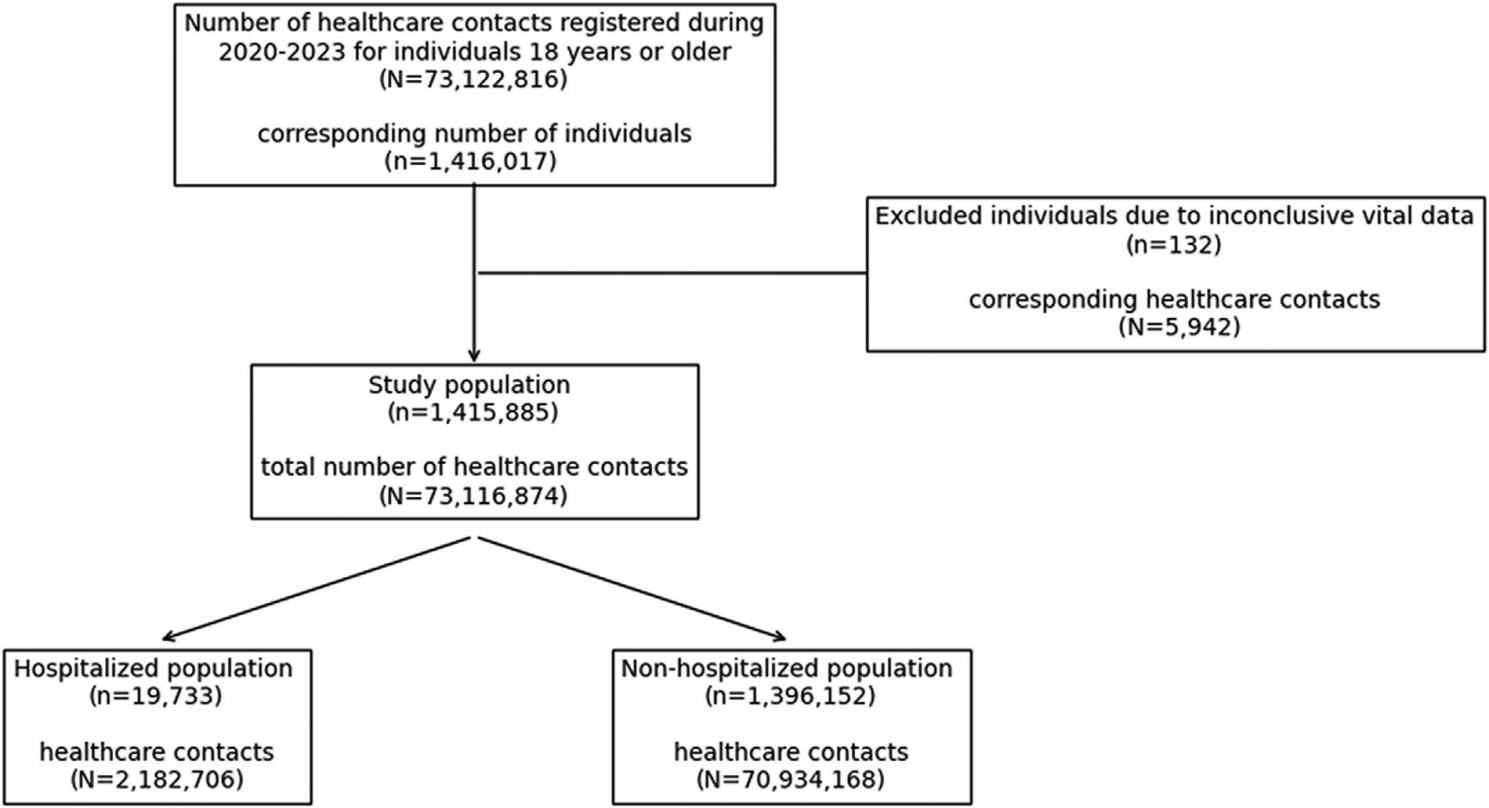
Flow chart of the healthcare contacts and individuals’ inclusion and exclusion, and subsequent distribution of the hospitalized and non‐hospitalized populations.

We included 1,415,885 individuals (Fig. 1), corresponding to >99% of the population 18 years and older in the Region Västra Götaland [10]. Of these, 9202 individuals (0.6%) had a recorded long COVID diagnosis. For 369,302 individuals, a record of an acute SARS‐CoV‐2 infection was available in the healthcare database; of these, 4391 (1.2%) individuals were identified as having long COVID ≥90 days after SARS‐CoV‐2. A total of 19,733 individuals had been hospitalized with a SARS‐CoV‐2 diagnosis (Fig. 1), and among those, 1574 had a long COVID diagnosis. A total of 1,388,524 individuals (>98% of the population 18 years and older in the region) had neither been hospitalized with a SARS‐CoV‐2 diagnosis nor had a recorded long COVID diagnosis. Thus, the no long COVID group in the non‐hospitalized population can essentially be considered the background population.

Baseline characteristics (before January 1, 2020) are shown in Table 1 and Table S3. For the non‐hospitalized population, the mean age was 48.5 for men and 47.2 for women. At baseline, in the non‐hospitalized population, obesity, asthma, depression, and anxiety were considerably more prevalent in the long COVID group compared with the no long COVID group in both men and women. In the hospitalized population, the long COVID group had a higher proportion of patients with severe infection (37.7% vs. 12.1% for men, 25.3% vs. 8.1% for women) (Table S3).

**Table 1:** Baseline characteristics (before January 1, 2020) of adult men and women in the Västra Götaland region by presence or absence of long COVID.

Individuals who had not been hospitalized with a SARS‐CoV‐2 infection *n* = 1,396,152						
	Men *n* = 686,381			Women *n* = 709,771		
	Long COVID *n* = 2559	No long COVID *n* = 683,822	*p*‐value	Long COVID *n* = 5069	No long COVID *n* = 704,702	*p*‐value
Age (years)	48.5 (14.4)	48.2 (19.0)	0.395	47.2 (13.7)	49.5 (19.9)	<0.001
18–39	714 (27.9)	264,449 (38.7)		1515 (29.9)	262,230 (37.2)	
40–59	1290 (50.4)	213,758 (31.3)		2707 (53.4)	211,488 (30.0)	
60–79	509 (19.9)	168,088 (24.6)		754 (14.9)	175,111 (24.8)	
80+	46 (1.8)	37,527 (5.5)		93 (1.8)	55,873 (7.9)	
Type 1 diabetes	40 (1.6)	10,618 (1.6)	1.000	44 (0.9)	7986 (1.1)	0.100
Type 2 diabetes	164 (6.4)	45,435 (6.6)	0.662	162 (3.2)	34,258 (4.9)	<0.001
Obesity	225 (8.8)	38,046 (5.6)	<0.001	524 (10.3)	54,573 (7.7)	<0.001
Dyslipidemia	336 (13.1)	92,141 (13.5)	0.631	386 (7.6)	82,426 (11.7)	<0.001
Hypertension	513 (20.0)	142,979 (20.9)	0.296	762 (15.0)	144,810 (20.5)	<0.001
Heart failure	39 (1.5)	16,065 (2.3)	0.007	33 (0.7)	13,691 (1.9)	<0.001
Atrial fibrillation	77 (3.0)	29,650 (4.3)	0.001	60 (1.2)	21,301 (3.0)	<0.001
Coronary heart disease	108 (4.2)	36,687 (5.4)	0.012	81 (1.6)	22,026 (3.1)	<0.001
Stroke	24 (0.9)	10,623 (1.6)	0.015	34 (0.7)	8658 (1.2)	<0.001
Peripheral vascular disease	8 (0.3)	3753 (0.5)	0.138	8 (0.2)	3476 (0.5)	0.001
Pulmonary embolism	17 (0.7)	3577 (0.5)	0.395	18 (0.4)	3543 (0.5)	0.167
Deep vein thrombosis	35 (1.4)	7661 (1.1)	0.275	65 (1.3)	10,077 (1.4)	0.410
COPD	36 (1.4)	13,297 (1.9)	0.058	75 (1.5)	17,284 (2.5)	<0.001
Asthma	225 (8.8)	37,984 (5.6)	<0.001	700 (13.8)	57,017 (8.1)	<0.001
Chronic kidney disease	32 (1.3)	13,156 (1.9)	0.016	21 (0.4)	10,231 (1.5)	<0.001
Dementia	6 (0.2)	6229 (0.9)	<0.001	16 (0.3)	10,124 (1.4)	<0.001
Depression	384 (15.0)	58,537 (8.6)	<0.001	1179 (23.3)	105,547 (15.0)	<0.001
Anxiety	447 (17.5)	70,004 (10.2)	<0.001	1490 (29.4)	129,576 (18.4)	<0.001

*Note*: Data as *n* (%) for categorical variables and mean (sd) for continuous variables.

Abbreviations: COPD, chronic obstructive pulmonary disease.

Tables 2 and 3 compare the prevalence of the relevant symptoms for the non‐hospitalized men and women in the long COVID and the no long COVID groups during the pre‐pandemic and pandemic periods, respectively. In the pre‐pandemic period, the prevalence of almost all the symptoms, except cognitive dysfunction and loss of smell/taste, was higher in the long COVID group than in the no long COVID group. A larger proportion of those in the long COVID group had healthcare contacts for any (one or more) of the relevant symptoms in the pre‐pandemic period, compared with the no long COVID group (57.6% vs. 36.3% for men and 71.6% vs. 50.4% for women). During the pandemic period, the difference was much more marked (88.8% vs. 41.6% for men and 92.9% vs. 55.9% for women). Similar results on symptom prevalence were seen in the hospitalized patients during the pandemic period (Table S5).

**Table 2:** Period prevalence during the pre‐pandemic (2016–2019) period of the relevant symptoms in men and women who had not been hospitalized with a SARS‐CoV‐2 infection.

	Men			Women		
	Long COVID *n* = 2559	No long COVID *n* = 683,822	*p*‐value	Long COVID *n* = 5069	No long COVID *n* = 704,702	*p*‐value
**Any of the symptoms**	1474 (57.6)	248,101 (36.3)	<0.001	3631 (71.6)	355,411 (50.4)	<0.001
**Cardiopulmonary**	795 (31.1)	121,017 (17.7)	<0.001	1986 (39.2)	178,796 (25.4)	<0.001
Dyspnea	188 (7.3)	30,938 (4.5)	<0.001	499 (9.8)	45,562 (6.5)	<0.001
Cough	641 (25.0)	90,328 (13.2)	<0.001	1509 (29.8)	128,302 (18.2)	<0.001
Palpitations	129 (5.0)	18,723 (2.7)	<0.001	479 (9.4)	41,958 (6.0)	<0.001
**CNS**	676 (26.4)	101,010 (14.8)	<0.001	2116 (41.7)	184,035 (26.1)	<0.001
Fatigue	492 (19.2)	62,171 (9.1)	<0.001	1651 (32.6)	125,944 (17.9)	<0.001
Cognitive dysfunction	15 (0.6)	5567 (0.8)	0.242	35 (0.7)	7100 (1.0)	0.029
Sleep disorder	271 (10.6)	45,023 (6.6)	<0.001	791 (15.6)	78,657 (11.2)	<0.001
Loss of smell/taste	4 (0.2)	979 (0.1)	1.000	12 (0.2)	1471 (0.2)	0.779
**Pain**	813 (31.8)	124,314 (18.2)	<0.001	2192 (43.2)	187,182 (26.6)	<0.001
Headache	264 (10.3)	35,863 (5.2)	<0.001	926 (18.3)	68,835 (9.8)	<0.001
Chest pain	327 (12.8)	49,723 (7.3)	<0.001	647 (12.8)	53,799 (7.6)	<0.001
Muscle/Joint pain	450 (17.6)	61,255 (9.0)	<0.001	1357 (26.8)	109,333 (15.5)	<0.001

*Note*: Data as *n* (%).

Abbreviation: CNS, central nervous system.

**Table 3:** Period prevalence during the pandemic (2020–2023) period of the relevant symptoms in men and women who had not been hospitalized with a SARS‐CoV‐2 infection.

	Men			Women		
	Long COVID *n* = 2559	No long COVID *n* = 683,822	*p*‐value	Long COVID *n* = 5069	No long COVID *n* = 704,702	*p*‐value
**Any of the symptoms**	2273 (88.8)	284,742 (41.6)	<0.001	4710 (92.9)	393,794 (55.9)	<0.001
**Cardiopulmonary**	1656 (64.7)	141,079 (20.6)	<0.001	3485 (68.8)	201,846 (28.6)	<0.001
Dyspnea	826 (32.3)	45,493 (6.7)	<0.001	1866 (36.8)	63,395 (9.0)	<0.001
Cough	1214 (47.4)	97,664 (14.3)	<0.001	2419 (47.7)	135,316 (19.2)	<0.001
Palpitations	301 (11.8)	23,411 (3.4)	<0.001	1018 (20.1)	50,768 (7.2)	<0.001
**CNS**	1504 (58.8)	137,190 (20.1)	<0.001	3502 (69.1)	225,480 (32.0)	<0.001
Fatigue	1259 (49.2)	85,728 (12.5)	<0.001	2984 (58.9)	155,632 (22.1)	<0.001
Cognitive dysfunction	46 (1.8)	10,124 (1.5)	0.214	88 (1.7)	11,898 (1.7)	0.835
Sleep disorder	490 (19.1)	60,608 (8.9)	<0.001	1242 (24.5)	97,075 (13.8)	<0.001
Loss of smell/taste	72 (2.8)	1977 (0.3)	<0.001	165 (3.3)	2899 (0.4)	<0.001
**Pain**	1172 (45.8)	129,430 (18.9)	<0.001	2894 (57.1)	193,868 (27.5)	<0.001
Headache	578 (22.6)	45,894 (6.7)	<0.001	1708 (33.7)	89,264 (12.7)	<0.001
Chest pain	547 (21.4)	56,515 (8.3)	<0.001	1110 (21.9)	62,833 (8.9)	<0.001
Muscle/Joint pain	458 (17.9)	50,141 (7.3)	<0.001	1357 (26.8)	89,570 (12.7)	<0.001

*Note*: Data as *n* (%).

Abbreviation: CNS, central nervous system.

By contrast, differences in symptom prevalence in the pre‐pandemic period were less pronounced in the hospitalized patients (Table S4), with the proportion of any symptom being only slightly higher in the long COVID group than the no long COVID group (62.6% vs. 60.6%, *p* = 0.394 for men; and 77.5% vs. 71.1%, *p* = 0.016 for women). Some symptoms were less prevalent in those who later developed long COVID. Findings were similar in the sensitivity analysis with long COVID ≥90 days after SARS‐CoV‐2 group (Tables S6–S9).

**Fig. 2:**
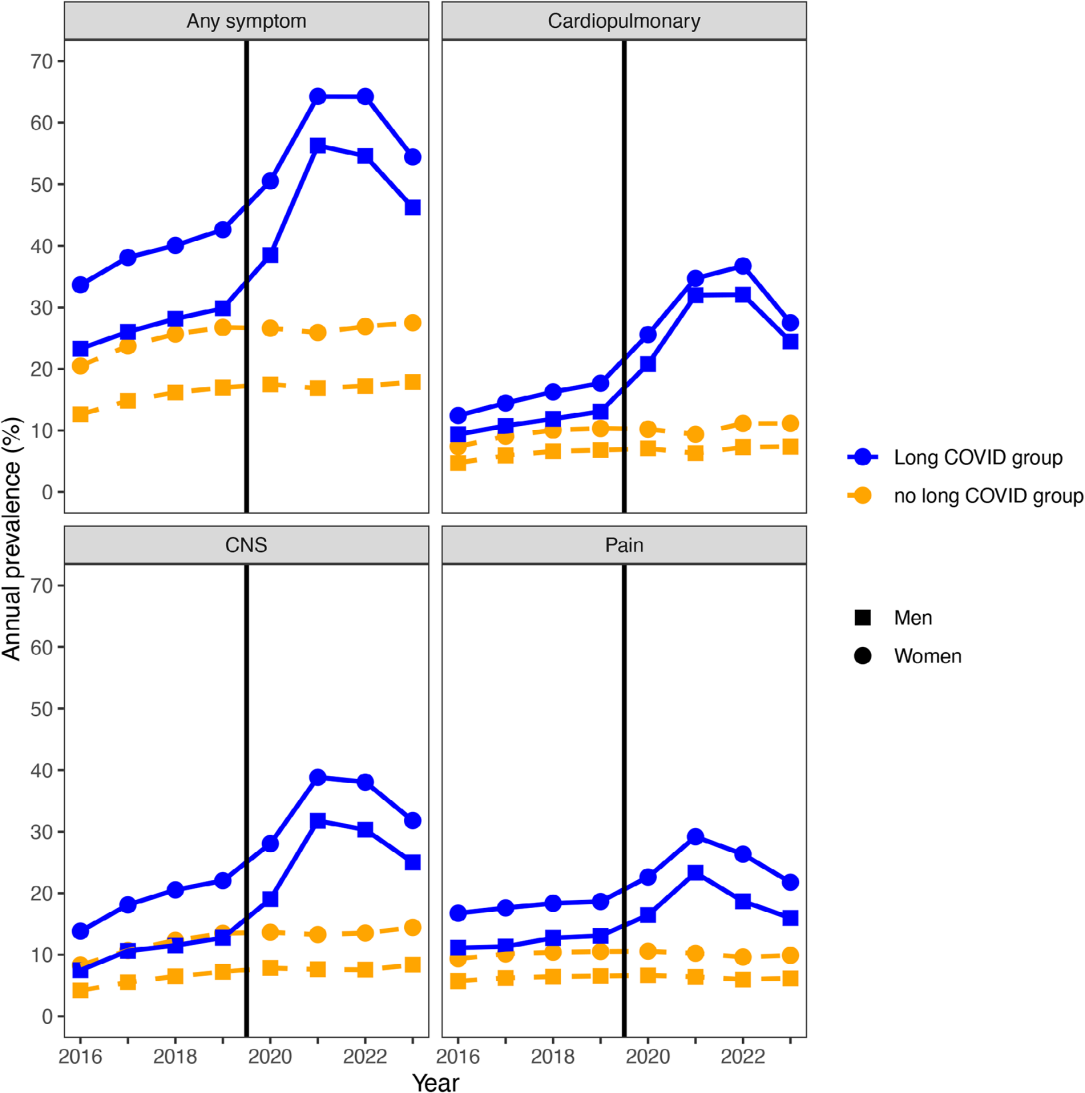
Annual prevalence of any (one or more) of the relevant symptoms overall and in each symptom category in men (squares) and women (circles) not hospitalized with a SARS‐CoV‐2 infection, comparing the long COVID group (blue, solid lines) versus the no long COVID group (orange, dashed lines). The black vertical line marks the transition from the pre‐pandemic period into the pandemic period.

**Fig. 3:**
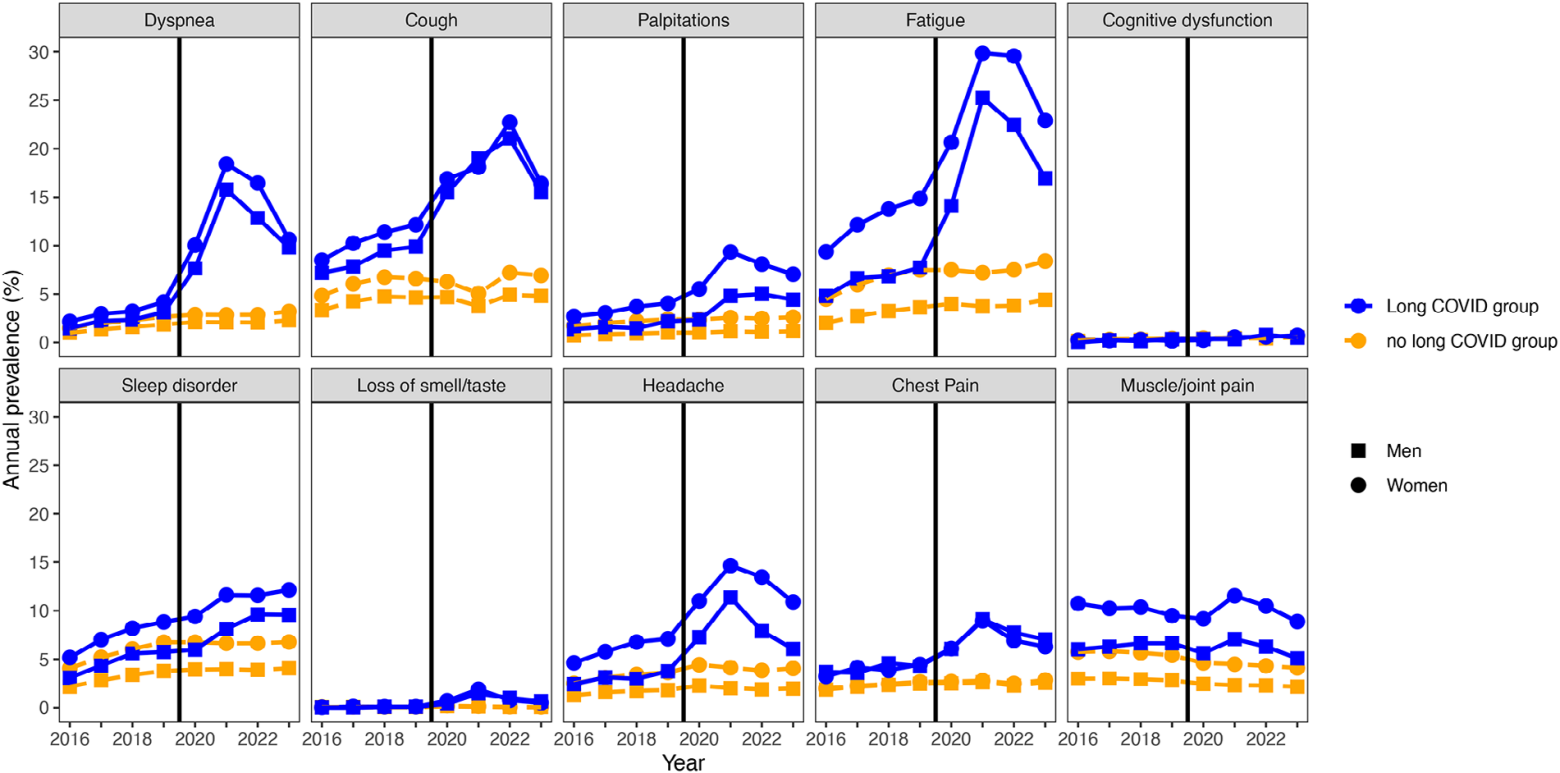
Annual prevalence of the relevant symptoms in men (squares) and women (circles) not hospitalized with a SARS‐CoV‐2 infection, comparing the long COVID group (blue, solid lines) versus the no long COVID group (orange, dashed lines). The black vertical line marks the transition from the pre‐pandemic period into the pandemic period.

Figs. 2 and 3 and Figs. S1 and S2 show the annual prevalence of the symptoms implicated in long COVID from 2016 to 2023 for the non‐hospitalized and hospitalized populations, respectively. For the non‐hospitalized population, the recorded prevalence of at least one of the symptoms overall and for each symptom category was markedly higher already during the pre‐pandemic period in the long COVID group, compared with the no long COVID group. From 2020, the annual prevalence of symptoms rose markedly in the long COVID group and then declined toward 2023. Among those in the no long COVID group, there was no corresponding rise in symptoms during 2020–2023, and the annual prevalence of recorded symptoms in the no long COVID group, accordingly, remained stable or decreased slightly. For the hospitalized population, the annual symptom prevalence of at least one symptom overall and for each symptom category was higher in the long COVID group than the no long COVID group during the pandemic period. By contrast, during the pre‐pandemic period, there was no marked difference between the long COVID group and no long COVID group for any of the symptom categories.

Table S10 shows the characteristics of the 60,092 individuals who died before the end of the study period. Of these, 220 (0.4%) had received a long COVID diagnosis.

**Fig. 4:**
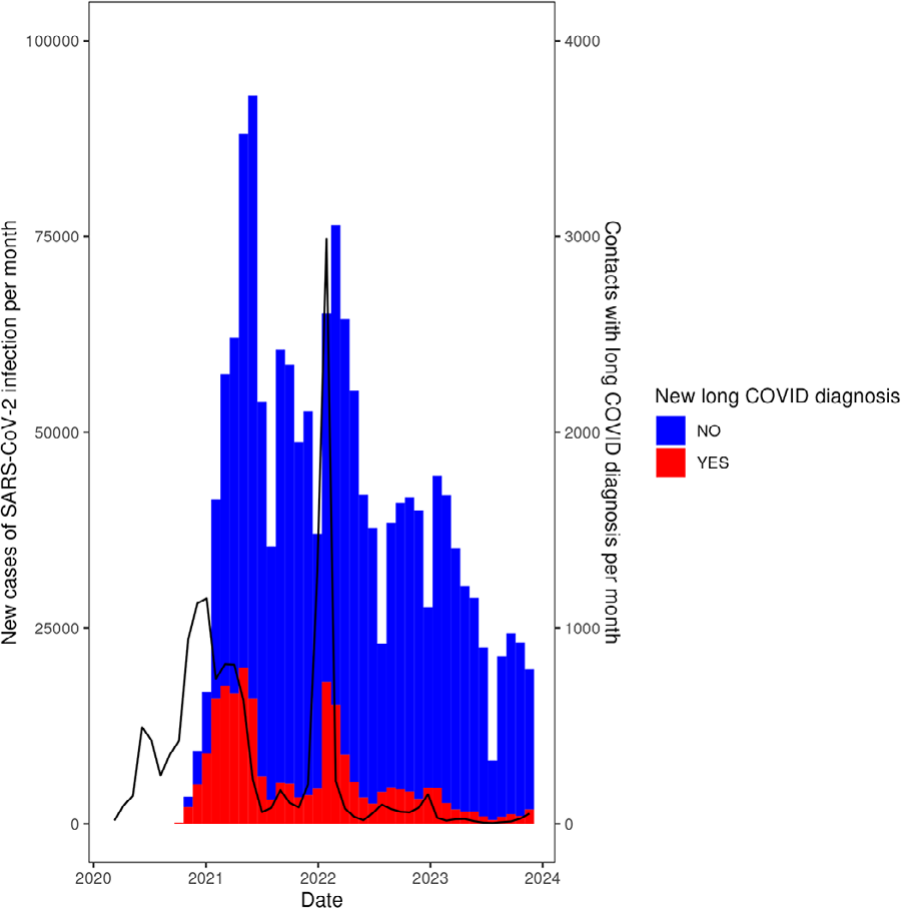
Number of healthcare contacts with a long COVID diagnosis per month (colored bars) and new patients with SARS‐CoV‐2 infection per month (black curved line) during the pandemic period. The red‐colored part of the bars represents the first time an individual patient is registered with a long COVID diagnosis. In other words, the red part of the bars represents the long COVID incidence.

Fig. 4 shows that there was a temporal association between long COVID incidence and the number of new patients with SARS‐CoV‐2 infections. There were two periods of peak PCC incidence: one early during the period from December 2020 to July 2021 and one late during the period from January to May 2022. Comparing the areas of the bars, we see that the early period comprised considerably more individuals than the late period. After the beginning of 2023, there was a marked decrease in the number of new patients with a long COVID diagnosis and SARS‐CoV‐2 infection. There was also a decrease in the total number of healthcare contacts with a long COVID diagnosis during the same period.

**Fig. 5:**
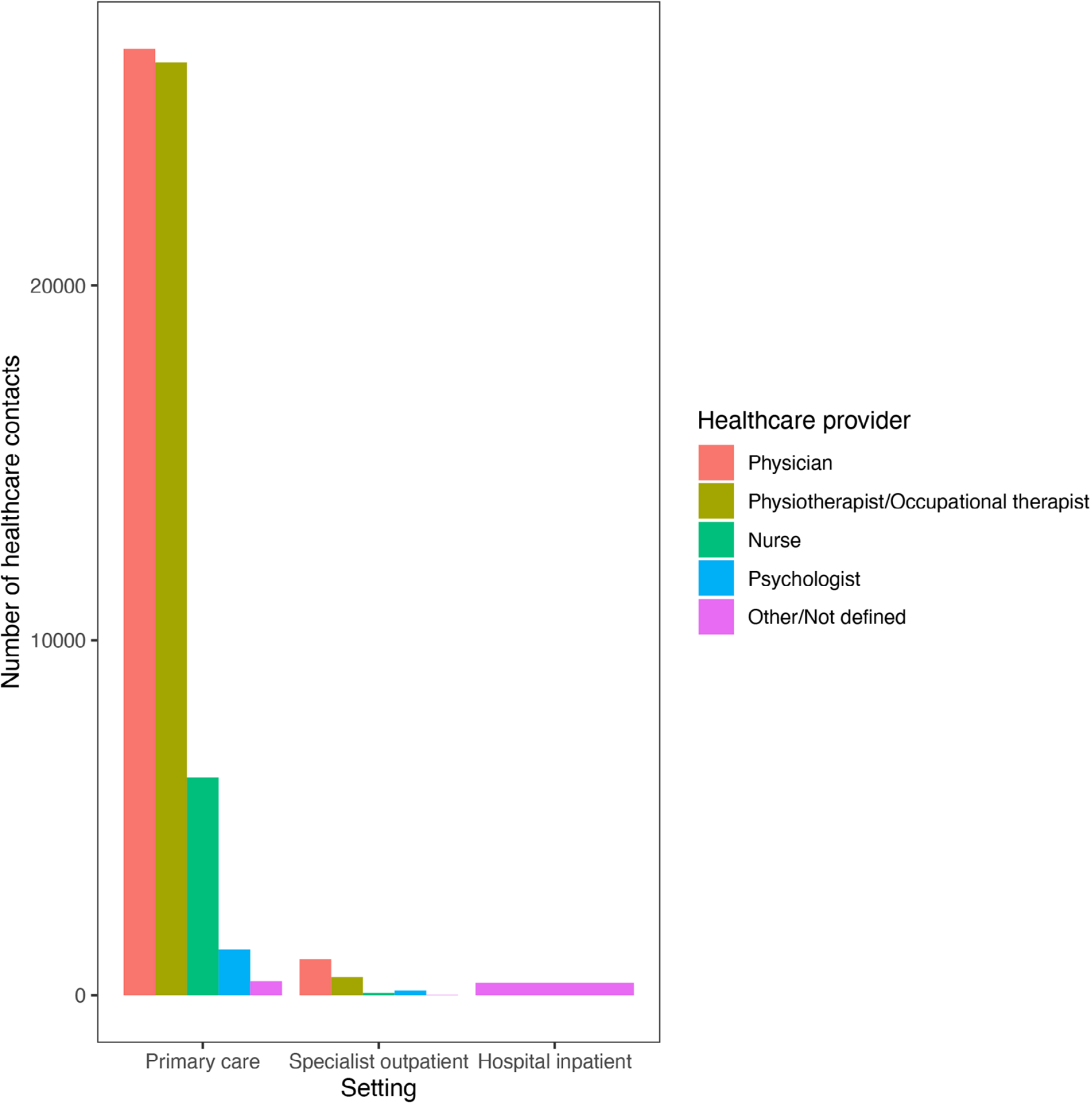
Number of healthcare contacts with a long COVID diagnosis during the study period, by type of healthcare provider and care setting. Specialist outpatient stands for specialist outpatient clinic or hospital outpatient clinic.

During the pandemic period (2020–2023), there was a total of 73,116,874 healthcare contacts registered for our study population (Fig. 1). Of these, 62,211 (0.09%) were registered with a long COVID diagnosis. In the non‐hospitalized population, this amounted to a mean of 6.8 healthcare contacts per patient with long COVID diagnosis for women and 5.1 for men; the median number of healthcare contacts per patient was two for both men and women (Table 4). Almost all the healthcare contacts (96.8%) took place in a primary care setting, with 44.0% of the healthcare contacts with a physician and 42.6% with a physiotherapist or occupational therapist (Fig. 5). The mean number of overall healthcare contacts over the period 2020–2023 was higher in the long COVID group compared with the no long COVID group (Table 5 and Table S11). In the non‐hospitalized population, the long COVID group had 89% and 84% more healthcare contacts than the no long COVID group for men and women, respectively. After adjusting for age group, obesity, asthma, and anxiety, the long COVID group had an average of 100% (95% CI 93%–107%) more healthcare contacts than the no long COVID group for men and 87% (95% CI 83%–92%) more for women.

**Table 4:** Healthcare contacts with a long COVID diagnosis.

	Non‐hospitalized		Hospitalized	
	Men	Women	Men	Women
Healthcare contacts	12,984	34,673	8619	5935
Mean per patient	5.1 (10.5)	6.8 (12.7)	9.5 (19.5)	9.0 (15.0)
Median per patient	2 (4)	2 (6)	3 (8)	3 (9)

*Note*: Data as *n*, mean (sd), median (upper quartile).

**Table 5:** Number of healthcare contacts overall during 2020–2023 in the non‐hospitalized population.

	Men		Women	
	Long COVID	No long COVID	Long COVID	No long COVID
Healthcare contacts	204,117	28,816,906	547,805	41,365,340
Mean per patient	79.8 (63.6)	42.1 (50.2)	108.1 (79.5)	58.7 (59.0)
Median per patient	61 (106)	26 (55)	89 (140)	41 (78)

*Note*: Data as *n*, mean (sd), median (upper quartile).

**Fig. 6:**
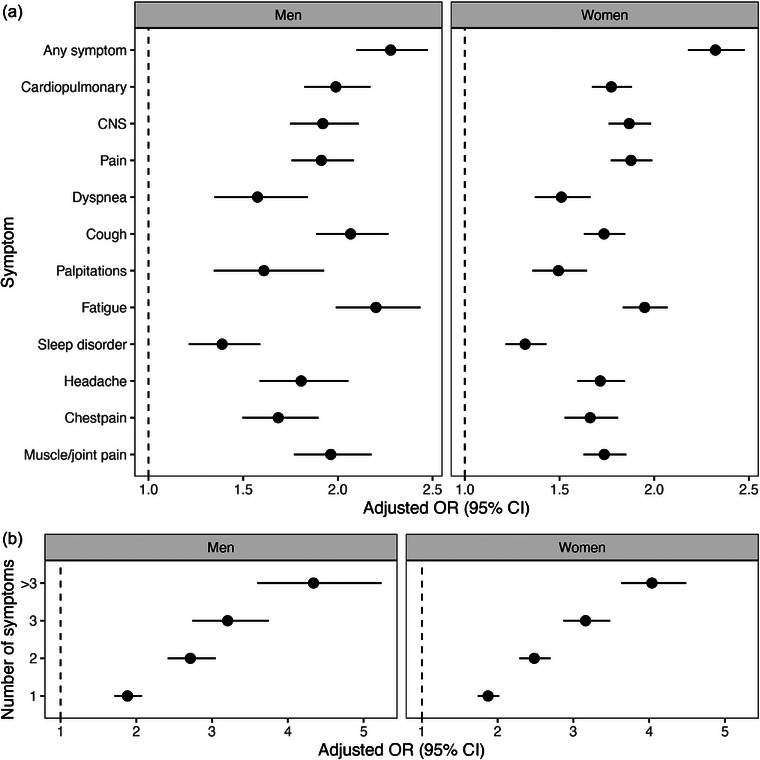
Multivariable logistic regressions of the association between the occurrence of relevant symptoms pre‐pandemically (a) or the number of symptoms (b) and receiving a long COVID diagnosis. Only the non‐hospitalized population is included. Each regression includes the symptom or symptom group of interest along with age group, obesity, asthma, and anxiety; that is the fully adjusted model. Not having a symptom (a) or zero symptoms (b) was used as a reference. CI, confidence interval; CNS, central nervous system; OR, odds ratio.

In the non‐hospitalized group, there was a modest correlation between depression and anxiety at baseline in both men and women (Figs. S3 and S4). Fig. 6 and Table S12 show that, in the non‐hospitalized group, having any of the relevant symptoms overall or in each symptom group pre‐pandemically was associated with receiving a long COVID diagnosis. Having any symptom resulted in an adjusted OR of 2.28 (95% CI 2.10–2.48) for men and 2.32 (95% CI 2.18–2.48) for women after adjusting for age group, obesity, asthma, and anxiety. The number of symptoms registered before the pandemic was also associated with receiving a long COVID diagnosis (Fig. 6, Table S13), with having more than three symptoms resulting in an adjusted OR of 4.34 (95% CI 3.60–5.23) for men and 4.04 (95% CI 3.63–4.49) for women after adjusting for age group, obesity, asthma, and anxiety.

Of note is that in the non‐hospitalized population, the long COVID group included a smaller proportion of very old individuals (<2%) than the no long COVID group (5.5% of men and 7.9% of women). After adjusting for age, there were no significant differences in the prevalence of most baseline comorbidities, such as heart failure, atrial fibrillation, coronary heart disease, stroke, and chronic kidney disease, between the groups (Table S14). By contrast, hypertension, asthma, obesity, depression, and anxiety at baseline were more often registered in the long COVID group than the no long COVID group, even after adjusting for age.

## Discussion

In this register‐based study with an almost complete coverage of all adults in a large Swedish region, we found that the period prevalence of long COVID was 0.6%. Most of the individuals with long COVID had not been hospitalized with a SARS‐CoV‐2 infection. Importantly, we found that, in the non‐hospitalized population, many of the relevant symptoms were already registered in the pre‐pandemic period in a considerably higher proportion of individuals in the long COVID group than in the no long COVID group. The presence of one or more of the relevant symptoms pre‐pandemically was associated with more than twofold odds of receiving a long COVID diagnosis, even after adjusting for confounders; and the more symptoms, the higher the odds. After the first few years of the pandemic period with an elevated level in the annual prevalence of the relevant symptoms and healthcare contacts for long COVID, 2023 showed a decrease in these numbers, possibly indicating recovery.

The proportion of patients with long COVID in the present study was within the lower range of estimates in previous reports, which have found numbers varying between 0.02% and 5% when long COVID was identified by ICD codes in electronic healthcare records [11, 12, 13, 14, 15, 16]. As most studies report the number of individuals with long COVID in a population of SARS‐CoV‐2‐infected individuals, we should compare these figures with 1.2%, which was the proportion of individuals with long COVID ≥90 days after SARS‐CoV‐2. In line with earlier research, this study found more women than men in the long COVID group and that hospitalization, in particular severe acute SARS‐CoV‐2 infection, was associated with long COVID [11, 12, 15, 17]. Individuals from the hospitalized population were more often men, older, and had more cardiovascular and metabolic comorbidities, which is in accordance with earlier studies [11, 12, 17].

Not many studies have been published on the prevalence of ICD‐10 codes of symptoms compatible with long COVID in the years before the pandemic. One study showed that patients with a confirmed SARS‐CoV‐2 infection had a slightly higher prevalence of symptoms in the years after the infection compared with before, without distinguishing between a long COVID and no long COVID group [18], which is in accordance with our findings. Our finding that relevant symptoms had a higher prevalence pre‐pandemically in non‐hospitalized patients with long COVID can be derived from the data presented in a recent study from Sweden that compared individuals with long COVID with matched controls prior to and following a SARS‐CoV‐2 infection [15]. Interestingly, the proportions of patients identified with prior diagnoses of the relevant symptoms in this recent study were considerably lower than in our study. Part of the explanation could be that this study specified symptom diagnoses with ICD‐10 codes to the third digit, whereas our study specifies some ICD‐10 codes only to the second digit and might therefore include more diagnoses. Another difference is the ICD‐10 codes used to define long COVID, where this study uses the codes U08.9 and U09.9, whereas we only used U09.9 because we considered U08.9 (personal history of COVID‐19, unspecified) not to be a diagnosis of long COVID. However, the main reason why we identified higher proportions of patients with the relevant symptoms is most likely that our study has data from primary care in addition to hospital and specialist outpatient care.

Even so, we found that the prevalence of loss of smell/taste and cognitive dysfunction was very low, which contrasts with previous studies [19, 20]. Likely, using ICD‐10 codes for identifying these symptoms results in a considerable underestimation. A recent study showed low sensitivity for capturing symptoms compatible with long COVID from ICD‐10 codes, in particular cognitive dysfunction [21]. Moreover, symptoms compatible with long COVID do not translate seamlessly to corresponding ICD‐10 codes. For example, post‐exertional malaise, which is a common symptom in patients with long COVID and described as different from regular fatigue [22], has, in our and in one other study [17], been included in the fatigue ICD‐10 code since there is no separate ICD‐10 code for this symptom. This might decrease the specificity of the symptom.

A Scottish study, in which participants were asked about symptoms experienced after a SARS‐CoV‐2 infection, found a higher prevalence than in our study but also found that many symptoms were common in the never‐infected individuals [23]. In our study, about one out of every two patients in the no long COVID group had at least one of the important symptoms during the pre‐pandemic period, with a slight increase during the pandemic period. That is, the occurrence of any of the relevant symptoms was common in the general population and not specific for long COVID.

The choice of symptoms to include in this study was based on expert opinion from the WHO (these were the symptoms that were identified as important to the clinical case definition of long COVID by more than 50% of the participants of the WHO Delphi consensus process). However, the importance of these symptoms has been contested by a study from the Netherlands with 76,422 participants, which empirically identified another set of 10 core symptoms (ageusia or anosmia, difficulties with breathing, chest pain, general tiredness, painful muscles, pain when breathing, lump in throat, heavy arms or legs, tingling extremities, and feeling hot and cold alternately) [24]. Five of these symptoms were included in the present study, whereas the remaining five had no ICD‐10 codes and were therefore beyond the scope of this study. Interestingly, general tiredness, rather than post‐exertional malaise, was identified as a core symptom.

The finding in the non‐hospitalized population that individuals in the long COVID group were more likely than individuals in the no long COVID group to have healthcare contacts for symptoms compatible with long COVID before the pandemic could indicate that individuals in the non‐hospitalized long COVID group are more vulnerable and thus more susceptible to suffering from many of the relevant symptoms due to other causes than the sequelae of a SARS‐CoV‐2 infection. This finding stands in agreement with a study from England showing that individuals with long COVID had more healthcare contacts than matched controls already in 2019 [25]. Interestingly, there was no clear difference in symptom prevalence pre‐pandemically in the hospitalized population, suggesting that hospitalized and non‐hospitalized individuals with long COVID likely represent different phenotypes.

The considerable overlap in symptom profiles between long COVID and encephalomyelitis/chronic fatigue syndrome is of note. It has been proposed that there could be a common etiopathogenesis for these post‐acute infectious syndromes [26]. A recent review article on post‐viral syndrome sheds light on our knowledge of persistent symptoms of pain, fatigue, and sleep disturbance after SARS‐CoV‐2 infection as well as other viral infections that were present before the SARS‐CoV‐2 pandemic, such as influenza B, Ebola, chikungunya, dengue, SARS, tick‐borne encephalitis, human immunodeficiency virus, polio, and varicella zoster virus [27]. The authors concluded that much remains unknown regarding the pathophysiology of post‐viral syndromes and that there is a need for improved understanding of post‐viral illness and how to help people cope with the sequelae.

The sensitivity analysis of the patients with long COVID ≥90 days after SARS‐CoV‐2 infection compared with patients who had a confirmed SARS‐CoV‐2 infection but no long COVID diagnosis 90 days after infection reproduced the above‐mentioned findings quite accurately. Although this study is not a validation study of the ICD‐10 code for long COVID, it contributes to the validity of the diagnosis. It should be noted that the WHO definition does not require a confirmed diagnosis of an acute SARS‐CoV‐2 infection, and it has been shown in an earlier study that symptom patterns are similar in patients with long COVID regardless of a prior infection [2]. There could be several reasons as to why there has been no registered SARS‐CoV‐2 infection for a patient with a long COVID diagnosis, such as a positive self‐test at home without a healthcare contact, a positive test outside of the Region Västra Götaland, a confirmed diagnosis in a family member, or symptoms compatible with a SARS‐CoV‐2 infection without a positive test.

In this study, we found two periods of peak long COVID incidence. The late long COVID period lasted for a shorter time and comprised fewer patients despite the preceding incidence peak of SARS‐CoV‐2 infections being higher. The reasons for this could be a decreasing trend of incident long COVID but also that the condition became less debilitating over time through increasing population immunity and the introduction of vaccines. COVID vaccination, starting in Sweden in December 2020, is a likely reason for the diminishing impact and incidence of long COVID [28], enhanced by repeated injections, as more doses result in lower risk for long COVID [29]. By December 2021, more than 2.9 million doses of vaccine had been administered in the Region Västra Götaland [30]. Thus, one of the major differences between individuals from the early and late long COVID incidence periods in our study was the vaccination coverage. Other factors could be differences depending on the strain of coronavirus causing the initial infection, with more recent strains having lower risk for debilitating long COVID [31]. Moreover, the healthcare system may have become more efficient over time at taking care of this group of patients. These topics are areas for future research.

Even though our study likely underestimates the actual long COVID prevalence in the population, it does account for the long COVID cases severe enough to have resulted in a healthcare contact. Moreover, healthcare contacts for the relevant symptoms did not increase beyond the baseline level in the no long COVID group, which indicates that few underdiagnosed patients with long COVID are hidden in the no long COVID group.

In this study, we found that the average number of overall healthcare contacts in the long COVID group was approximately twice that of the no long COVID group. Although the difference in our study was larger, it is in accordance with recent studies showing that individuals with long COVID have more overall healthcare utilization than matched controls [12, 25]. Although long COVID provides an extra burden to the healthcare system, we also see that the annual prevalence of relevant symptoms, the monthly number of healthcare contacts for long COVID, and the number of new patients with this diagnosis are all declining. Therefore, if this trend continues, the impact of long COVID on the Swedish healthcare system will be limited.

A strength of this study is that it has nearly complete (>99%) coverage of the population from a large region in Sweden, with comprehensive data from all healthcare providers in the region, both public and private, with individual‐level data spanning several years preceding as well as following the start of the COVID‐19 pandemic. The fact that the overwhelming number of contacts for long COVID occurred in primary care emphasizes the importance of including data from primary care when studying long COVID. However, there are also several limitations, such as, for example, that data on socio‐economic status was not available in this dataset, which has been shown in earlier studies to be associated with long COVID [11, 23].

One of the limitations of this study is the lack of information on how long the individuals had been residing in the Region Västra Götaland. Migration into and out of the region could thus not be considered. Moreover, there was no adjustment for the reduced follow‐up time for individuals who died during the period. Furthermore, because the long COVID group was defined without taking the exact time of diagnosis into account, the annual prevalences of symptoms will to some extent include individuals who at that time do not have a long COVID diagnosis in the denominator. Thus, the results from the pandemic period need to be interpreted with some caution. The data on annual prevalences show how common the symptoms are from the perspective of the healthcare system. We decided to use the same fixed denominator in both the pandemic and pre‐pandemic periods, the rationale being that we are comparing individuals who belong to either the long COVID group or no long COVID group, rather than individuals who already have been diagnosed with long COVID with those who have not.

A general limitation of using the ICD‐10 code U09.9 to identify long COVID is that it has not been validated in a Swedish setting. Although the sensitivity and specificity of the ICD‐10 code U09.9 for the identification of long COVID has been questioned in studies from the United States [17, 21], one study from Northern Denmark demonstrated that the U09.9 code had a high positive predictive value for long COVID and concluded it to be suitable for future registry‐based studies [32].

In conclusion, many of the relevant symptoms in long COVID were more prevalent in the long COVID group than in the no long COVID group during the pandemic. Although being registered with any of the symptoms was quite common in the population at large, there was a considerable correlation between having had a healthcare contact for any of the symptoms and later receiving a diagnosis of long COVID in the non‐hospitalized population. The number of healthcare contacts for long COVID in the Swedish healthcare system has been manageable. Although long COVID can significantly impact the quality of life for some individuals, the impact of long COVID on the healthcare system is declining, as evidenced by the decline in long COVID incidence and annual prevalence in symptoms relevant to long COVID.

## Author contributions

Vincent Lak and Annika Rosengren conceptualized the study. Vincent Lak, Annika Rosengren, Helen Sjöland, and Martin Adiels contributed to the data curation. Vincent Lak performed the formal analysis, producing the tables and figures and statistical analyses. Vincent Lak conducted literature search and wrote the original draft of the report, and Annika Rosengren provided supervision. Martin Adiels verified the underlying data. All authors provided critical review and commentary and contributed to the revision of the article manuscript. All authors had access to the underlying data and accept the responsibility to submit.

## Conflict of interest statement

The authors declare no conflicts of interest.

## Funding information

This work was supported by grants from the Swedish state under an agreement between the Swedish government and the county councils (ALF agreement) ALFGBG‐966211 (A.R.), ALFGBG‐942707 (J.R.), ALFGBG‐971608 (M.L.), ALFGBG‐943008 (H.S.), ALFGBG‐1006439 and The Swedish Research Council [VRREG 2019‐00193, 2020–05792, 2021–06525, 2023‐02144] (A.R.), the Swedish Heart and Lung Foundation 2021‐0345 and 2024‐0678 (A.R.), the Swedish Research Council for Health, Working Life and Welfare (2021‐00304) (M.Å.). The funders had no role in any aspect of the study design, data collection, analysis and interpretation of data, or writing of the report.

## Supporting information




**Table S1**. Categorization of important PCC symptoms, the naming of symptoms in this study and the corresponding WHO symptom names and ICD‐10 codes.
**Table S2**. ICD‐10 codes for baseline comorbidities.
**Table S3**. Baseline characteristics of men and women who had been hospitalized with a SARS‐CoV‐2 infection, and number of patients hospitalized with severe infection.
**Table S4**. Period prevalence during the pre‐pandemic (2016‐2019) period of the relevant symptoms in men and women who had been hospitalized with a SARS‐CoV‐2 infection.
**Table S5**. Period prevalence during the pandemic (2020‐2023) period of the relevant symptoms in men and women who had been hospitalized with a SARS‐CoV‐2 infection.
**Table S6**. Period prevalence during the pre‐pandemic (2016‐2019) period of the relevant symptoms in men and women with a record of an acute SARS‐CoV‐2 infection who had not been hospitalized with a SARS‐CoV‐2 infection comparing the long COVID ≥ 90 days after group with the no long COVID ≥ 90 days after group.
**Table S7**. Period prevalence during the pandemic (2020‐2023) period of the relevant symptoms in men and women with a record of an acute SARS‐CoV‐2 infection who had not been hospitalized with a SARS‐CoV‐2 infection comparing the long COVID ≥ 90 days after group with the no long COVID ≥ 90 days after group.
**Table S8**. Period prevalence during the pre‐pandemic (2016‐2019) period of the relevant symptoms in men and women with a record of an acute SARS‐CoV‐2 infection who had been hospitalized with a SARS‐CoV‐2 infection comparing the long COVID ≥ 90 days after group with the no long COVID ≥ 90 days after group.
**Table S9**. Period prevalence during the pandemic (2020‐2023) period of the relevant symptoms in men and women with a record of an acute SARS‐CoV‐2 infection who had been hospitalized with a SARS‐CoV‐2 infection comparing the long COVID ≥ 90 days after group with the no long COVID ≥ 90 days after group.
**Table S10**. Characteristics of the individuals who died before the end of the study period.
**Table S11**. Number of healthcare contacts overall during 2020‐2023 in the hospitalized population.
**Table S12**. Adjusted OR from logistic regression models of association between occurrence of symptoms pre‐pandemically and receiving a long COVID diagnosis in men and women in the non‐hospitalized population. Univariate model and the multivariable models are used. The multivariable models include age group, obesity, asthma and anxiety in addition to the symptom of interest.
**Table S13**. Adjusted OR from logistic regression models of association between number of symptoms pre‐pandemically and receiving a long COVID diagnosis in men and women in the non‐hospitalized population. Number of symptoms was categorized into zero, one, two, three and more than three symptoms. Having zero symptoms was used as reference. Univariate model and the multivariable models are used. The multivariable models include age group, obesity, asthma and anxiety in addition to the number of symptoms.
**Table S14**. Odds ratios (OR) and 95% confidence intervals (CI) for baseline comorbidities using logistic regression with no Long COVID as reference. The unadjusted odds ratio is derived from a univariate model. The adjusted model is a bivariate model with age added as a dependent variable.
**Figure S1**. Annual prevalence of any (one or more) of the important symptoms overall and in each symptom category in men (squares) and women (circles) hospitalized with a SARS‐CoV‐2 infection, comparing the long COVID group (blue, solid lines) vs the no long COVID group (orange, dashed lines). The black vertical line marks the transition from the pre‐pandemic period into the pandemic period.
**Figure S2**. Annual prevalence of the important symptoms in men (squares) and women (circles) hospitalized with a SARS‐CoV‐2 infection, comparing the long COVID group (blue, solid lines) vs the no long COVID group (orange, dashed lines). The black vertical line marks the transition from the pre‐pandemic period into the pandemic period.
**Figure S3**. Correlation matrix of the baseline comorbidities with a count of 100 or more individuals in the long COVID group that had statistically significant difference in prevalence compared with the no long COVID group in both men and women. The plot looks identical for men and women. Size and color of the circles corresponds to the magnitude of the correlation (larger and darker color represents a stronger correlation).

## Data Availability

Data are available from the VEGA database on request to the data providers, fulfilling legal and regulatory requirements and with permission from the Swedish Ethical Review Authority [9].
