# Supplementary material for: Preexisting symptoms increase the risk of developing long COVID during the SARS‐CoV‐2 pandemic
Source: J Intern Med. 2025 Jun 4;298(2):107–22. doi: 10.1111/joim.20102 (PMC12239058; doi:10.1111/joim.20102)
Supplement: Supplementary file 1 — Table S1. Categorization of important PCC symptoms, the naming of symptoms in this study and the corresponding WHO symptom names and ICD‐10 codes. Table S2. ICD‐10 codes for baseline comorbidities. Table S3. Baseline characteristics of men and women who had been hospitalized with a SARS‐CoV‐2 infection, and number of patients hospitalized with severe infection. Table S4. Period prevalence during the pre‐pandemic (2016‐2019) period of the relevant symptoms in men and women who had been hospitalized with a SARS‐CoV‐2 infection. Table S5. Period prevalence during the pandemic (2020‐2023) period of the relevant symptoms in men and women who had been hospitalized with a SARS‐CoV‐2 infection. Table S6. Period prevalence during the pre‐pandemic (2016‐2019) period of the relevant symptoms in men and women with a record of an acute SARS‐CoV‐2 infection who had not been hospitalized with a SARS‐CoV‐2 infection comparing the long COVID ≥ 90 days after group with the no long COVID ≥ 90 days after group. Table S7. Period prevalence during the pandemic (2020‐2023) period of the relevant symptoms in men and women with a record of an acute SARS‐CoV‐2 infection who had not been hospitalized with a SARS‐CoV‐2 infection comparing the long COVID ≥ 90 days after group with the no long COVID ≥ 90 days after group. Table S8. Period prevalence during the pre‐pandemic (2016‐2019) period of the relevant symptoms in men and women with a record of an acute SARS‐CoV‐2 infection who had been hospitalized with a SARS‐CoV‐2 infection comparing the long COVID ≥ 90 days after group with the no long COVID ≥ 90 days after group. Table S9. Period prevalence during the pandemic (2020‐2023) period of the relevant symptoms in men and women with a record of an acute SARS‐CoV‐2 infection who had been hospitalized with a SARS‐CoV‐2 infection comparing the long COVID ≥ 90 days after group with the no long COVID ≥ 90 days after group. Table S10. Characteristics of the individuals who died before the end of the [file JOIM-298-107-s001.docx]

## Supplementary information

Preexisting symptoms increase the risk of developing long COVID during the SARS-CoV-2 pandemic

Vincent Laka,b, Helen Sjölanda,b, Martin Adielsa,c, Christina E Lundberga,d, Josefina Robertsone,f,g, Maria Åberge,h, Christian Alexa,b, Martin Lindgrena,b, Annika Rosengrena,b

a Department of Molecular and Clinical Medicine, Institute of Medicine, Sahlgrenska Academy, University of Gothenburg, Gothenburg, Sweden.

b Department of Medicine Geriatrics and Emergency Medicine, Sahlgrenska University Hospital, Östra Hospital, Region Västra Götaland, Gothenburg, Sweden.

c School of Public Health and Community Medicine, Institute of Medicine, University of Gothenburg, Gothenburg, Sweden.

d Department of Food and Nutrition and Sport Science, Faculty of Education, University of Gothenburg, Gothenburg, Sweden.

e General Practice/Family Medicine, School of Public Health and Community Medicine, Institute of Medicine, Sahlgrenska Academy, University of Gothenburg, Gothenburg, Sweden.

f Department of Infectious Diseases, Institute of Biomedicine, Sahlgrenska Academy, University of Gothenburg, Gothenburg, Sweden.

g Department of Infectious Diseases, Sahlgrenska University Hospital, Region Västra Götaland, Gothenburg, Sweden.

h Region Västra Götaland, Regionhälsan, Gothenburg, Sweden.

## **Table of contents**

## **Appendix A· Supplementary data**

## **Supplementary tables 3**

## **Supplementary figures 13**

## **Appendix A· Supplementary data**

**S table 1.** Categorization of important PCC symptoms, the naming of symptoms in this study and the corresponding WHO symptom names and ICD-10 codes.

| **Category** | **Symptom name** | **WHO symptom names*** | **ICD-10 code** |
| --- | --- | --- | --- |
|  |  |  |  |
| Cardiopulmonary: | Dyspnea | Shortness of breath | R060 |
|  | Cough | Cough | R05 |
|  | Palpitations | Tachycardia | R000, R002, R008 |
|  |  |  |  |
| Central nervous system: | Fatigue | Fatigue,  Post-exertional malaise | R53 |
|  | Cognitive dysfunction | Cognitive dysfunction,  Impaired memory | R41 |
|  |  |  |  |
|  | Sleep disorder | Sleep disorders | G479 |
|  | Loss of smell/taste | Altered smell/taste | R43 |
|  |  |  |  |
| Pain: | Headache | Headache | R51 |
|  | Chest pain | Chest pain | R07 |
|  | Muscle/joint pain | Joint pain,  Muscle spasms | M791, M255 |

* Symptom names are as listed in Appendix 5, domain 5 of the WHO clinical case definition document ^1^.

**S table 2.** ICD-10 codes for baseline comorbidities.

| **Comorbidity** | **ICD-10 code** |
| --- | --- |
| Diabetes type 1 | E10 |
| Diabetes type 2 | E11 |
| Obesity | E66 |
| Dyslipidemia | E78 |
| Hypertension | I10-I15 |
| Heart failure (HF) | I50 |
| Atrial fibrillation (AF) | I48 |
| Coronary heart disease (CHD) | I20, I21, I25 |
| Stroke | I61, I63 |
| Peripheral vascular disease (PVD) | I739 |
| Pulmonary embolism (PE) | I26 |
| Deep vein thrombosis (DVT) | I80 |
| Chronic obstructive pulmonary disease (COPD) | J44 |
| Chronic kidney disease | N18, N19 |
| Asthma | J45 |
| Dementia | F00-F03, G30 |
| Depression | F32-F34 |
| Anxiety | F41 |

**S table 3.** Baseline characteristics of men and women who had been hospitalized with a SARS-CoV-2 infection, and number of patients hospitalized with severe infection.

| Individuals who had been hospitalized with a SARS-CoV-2 infection  n = 19,733 | | | | | | |
| --- | --- | --- | --- | --- | --- | --- |
|  | Men  n = 11,226 | |  | Women  n = 8,507 | |  |
|  | Long COVID  n = 911 | No long COVID  n = 10,315 | p-value | Long COVID  n = 663 | No long COVID  n = 7,844 | p-value |
| Severe infection | 343 (37.7) | 1,251 (12.1) | <0.001 | 168 (25.3) | 633 (8.1) | <0.001 |
| Age (years) | 60.8 (13.8) | 68.0 (16.7) | <0.001 | 58.4 (16.0) | 67.6 (19.6) | <0.001 |
| 18-39 | 52 (5.7) | 774 (7.5) |  | 78 (11.8) | 1,048 (13.4) |  |
| 40-59 | 373 (40.9) | 2,065 (20.0) |  | 309 (46.6) | 1,239 (15.8) |  |
| 60-79 | 398 (43.7) | 4,604 (44.6) |  | 198 (29.9) | 2,938 (37.5) |  |
| 80+ | 88 (9.7) | 2,872 (27.8) |  | 78 (11.8) | 2,619 (33.4) |  |
| Type_1 Diabetes | 33 (3.6) | 547 (5.3) | 0.034 | 14 (2.1) | 309 (3.9) | 0.024 |
| Type_2 Diabetes | 182 (20.0) | 2,459 (23.8) | 0.010 | 93 (14.0) | 1,559 (19.9) | <0.001 |
| Obesity | 176 (19.3) | 1,497 (14.5) | <0.001 | 168 (25.3) | 1,353 (17.2) | <0.001 |
| Dyslipidemia | 271 (29.7) | 3,767 (36.5) | <0.001 | 129 (19.5) | 2,440 (31.1) | <0.001 |
| Hypertension | 415 (45.6) | 5,785 (56.1) | <0.001 | 270 (40.7) | 4,355 (55.5) | <0.001 |
| Heart failure | 50 (5.5) | 1,423 (13.8) | <0.001 | 46 (6.9) | 1,033 (13.2) | <0.001 |
| Atrial fibrillation | 65 (7.1) | 1,873 (18.2) | <0.001 | 41 (6.2) | 1,114 (14.2) | <0.001 |
| Coronary heart disease | 117 (12.8) | 2,221 (21.5) | <0.001 | 39 (5.9) | 1,195 (15.2) | <0.001 |
| Stroke | 37 (4.1) | 759 (7.4) | <0.001 | 17 (2.6) | 444 (5.7) | 0.001 |
| Peripheral vascular disease | 12 (1.3) | 304 (2.9) | 0.006 | 9 (1.4) | 181 (2.3) | 0.146 |
| Pulmonary embolism | 15 (1.6) | 185 (1.8) | 0.849 | 7 (1.1) | 176 (2.2) | 0.059 |
| Deep vein thrombosis | 31 (3.4) | 326 (3.2) | 0.763 | 31 (4.7) | 302 (3.9) | 0.343 |
| COPD | 54 (5.9) | 978 (9.5) | <0.001 | 49 (7.4) | 909 (11.6) | 0.001 |
| Asthma | 109 (12.0) | 935 (9.1) | 0.005 | 145 (21.9) | 1,175 (15.0) | <0.001 |
| Chronic kidney disease | 49 (5.4) | 1,179 (11.4) | <0.001 | 36 (5.4) | 729 (9.3) | 0.001 |
| Dementia | 8 (0.9) | 506 (4.9) | <0.001 | 8 (1.2) | 438 (5.6) | <0.001 |
| Depression | 127 (13.9) | 1,510 (14.6) | 0.601 | 139 (21.0) | 1,747 (22.3) | 0.466 |
| Anxiety | 126 (13.8) | 1,452 (14.1) | 0.877 | 184 (27.8) | 2,003 (25.5) | 0.227 |

Data as n (%) for categorical variables and mean (sd) for continuous variables.

Severe infection defined as requiring mechanical ventilation or high-flow oxygen therapy.

COPD: Chronic obstructive pulmonary disease.

**S table 4.** Period prevalence during the pre-pandemic (2016-2019) period of the relevant symptoms in men and women who had been hospitalized with a SARS-CoV-2 infection.

|  | Men | | | Women | | |
| --- | --- | --- | --- | --- | --- | --- |
|  | Long COVID  n = 911 | No long COVID  n = 10,315 | p-value | Long COVID  n = 663 | No long COVID  n = 7,844 | p-value |
| **Any of the symptoms** | 566 (62.1) | 6,254 (60.6) | 0.394 | 501 (75.6) | 5,575 (71.1) | 0.016 |
| **Cardiopulmonary** | 329 (36.1) | 3,526 (34.2) | 0.254 | 312 (47.1) | 3,327 (42.4) | 0.023 |
| Dyspnea | 113 (12.4) | 1,600 (15.5) | 0.014 | 118 (17.8) | 1,599 (20.4) | 0.123 |
| Cough | 248 (27.2) | 2,439 (23.6) | 0.017 | 239 (36.0) | 2,175 (27.7) | <0.001 |
| Palpitations | 40 (4.4) | 394 (3.8) | 0.443 | 59 (8.9) | 681 (8.7) | 0.905 |
| **CNS** | 261 (28.6) | 3,155 (30.6) | 0.238 | 297 (44.8) | 3,223 (41.1) | 0.069 |
| Fatigue | 160 (17.6) | 1,859 (18.0) | 0.763 | 213 (32.1) | 2,019 (25.7) | <0.001 |
| Cognitive dysfunction | 14 (1.5) | 427 (4.1) | <0.001 | 8 (1.2) | 321 (4.1) | <0.001 |
| Sleep disorder | 125 (13.7) | 1,474 (14.3) | 0.674 | 137 (20.7) | 1,661 (21.2) | 0.795 |
| Loss of smell/taste | 2 (0.2) | 14 (0.1) | 0.853 | 2 (0.3) | 15 (0.2) | 0.874 |
| **Pain** | 315 (34.6) | 3,131 (30.4) | 0.009 | 331 (49.9) | 3,156 (40.2) | <0.001 |
| Headache | 85 (9.3) | 761 (7.4) | 0.038 | 126 (19.0) | 1,008 (12.9) | <0.001 |
| Chest pain | 143 (15.7) | 1,581 (15.3) | 0.803 | 134 (20.2) | 1,292 (16.5) | 0.015 |
| Muscle/joint pain | 178 (19.5) | 1,611 (15.6) | 0.002 | 211 (31.8) | 1,904 (24.3) | <0.001 |

Data as n (%).

**S table 5.** Period prevalence during the pandemic (2020-2023) period of the relevant symptoms in men and women who had been hospitalized with a SARS-CoV-2 infection.

|  | Men | | | Women | | |
| --- | --- | --- | --- | --- | --- | --- |
|  | Long COVID  n = 911 | No long COVID  n = 10,315 | p-value | Long COVID  n = 663 | No long COVID  n = 7,844 | p-value |
| **Any of the symptoms** | 822 (90.2) | 8,056 (78.1) | <0.001 | 627 (94.6) | 6,426 (81.9) | <0.001 |
| **Cardiopulmonary** | 676 (74.2) | 5,372 (52.1) | <0.001 | 536 (80.8) | 4,333 (55.2) | <0.001 |
| Dyspnea | 543 (59.6) | 3,757 (36.4) | <0.001 | 411 (62.0) | 3,039 (38.7) | <0.001 |
| Cough | 408 (44.8) | 2,860 (27.7) | <0.001 | 362 (54.6) | 2,265 (28.9) | <0.001 |
| Palpitations | 81 (8.9) | 481 (4.7) | <0.001 | 126 (19.0) | 700 (8.9) | <0.001 |
| **CNS** | 531 (58.3) | 4,917 (47.7) | <0.001 | 460 (69.4) | 4,281 (54.6) | <0.001 |
| Fatigue | 406 (44.6) | 3,361 (32.6) | <0.001 | 380 (57.3) | 2,901 (37.0) | <0.001 |
| Cognitive dysfunction | 56 (6.1) | 1,015 (9.8) | <0.001 | 31 (4.7) | 763 (9.7) | <0.001 |
| Sleep disorder | 220 (24.1) | 1,974 (19.1) | <0.001 | 187 (28.2) | 1,994 (25.4) | 0.126 |
| Loss of smell/taste | 11 (1.2) | 42 (0.4) | 0.002 | 14 (2.1) | 32 (0.4) | <0.001 |
| **Pain** | 374 (41.1) | 3,086 (29.9) | <0.001 | 380 (57.3) | 2,942 (37.5) | <0.001 |
| Headache | 153 (16.8) | 897 (8.7) | <0.001 | 183 (27.6) | 1,131 (14.4) | <0.001 |
| Chest pain | 185 (20.3) | 1,808 (17.5) | 0.039 | 184 (27.8) | 1,379 (17.6) | <0.001 |
| Muscle/joint pain | 160 (17.6) | 1,138 (11.0) | <0.001 | 199 (30.0) | 1,338 (17.1) | <0.001 |

Data as n (%).

**S table 6.** Period prevalence during the pre-pandemic (2016-2019) period of the relevant symptoms in men and women with a record of an acute SARS-CoV-2 infection who had not been hospitalized with a SARS-CoV-2 infection comparing the long COVID ≥ 90 days after group with the no long COVID ≥ 90 days after group.

|  | Men | | | Women | | |
| --- | --- | --- | --- | --- | --- | --- |
|  | Long COVID ≥ 90 days  n = 1,011 | No long COVID  ≥ 90 days  n = 159,293 | p-value | Long COVID  ≥ 90 days  n = 2,370 | No long COVID  ≥ 90 days  n = 186,895 | p-value |
| **Any of the symptoms** | 614 (60.7) | 62,023 (38.9) | <0.001 | 1,789 (75.5) | 102,745 (55.0) | <0.001 |
| **Cardiopulmonary** | 334 (33.0) | 30,730 (19.3) | <0.001 | 992 (41.9) | 51,576 (27.6) | <0.001 |
| Dyspnea | 79 (7.8) | 7,060 (4.4) | <0.001 | 259 (10.9) | 12,145 (6.5) | <0.001 |
| Cough | 264 (26.1) | 23,823 (15.0) | <0.001 | 762 (32.2) | 38,608 (20.7) | <0.001 |
| Palpitations | 56 (5.5) | 4,629 (2.9) | <0.001 | 240 (10.1) | 11,482 (6.1) | <0.001 |
| **CNS** | 293 (29.0) | 25,091 (15.8) | <0.001 | 1,046 (44.1) | 54,448 (29.1) | <0.001 |
| Fatigue | 218 (21.6) | 16,440 (10.3) | <0.001 | 826 (34.9) | 40,233 (21.5) | <0.001 |
| Cognitive dysfunction | 9 (0.9) | 1,323 (0.8) | 0.972 | 16 (0.7) | 2,006 (1.1) | 0.076 |
| Sleep disorder | 118 (11.7) | 10,456 (6.6) | <0.001 | 400 (16.9) | 20,743 (11.1) | <0.001 |
| Loss of smell/taste | 2 (0.2) | 224 (0.1) | 0.950 | 9 (0.4) | 383 (0.2) | 0.102 |
| **Pain** | 350 (34.6) | 32,288 (20.3) | <0.001 | 1,070 (45.1) | 56,494 (30.2) | <0.001 |
| Headache | 123 (12.2) | 10,393 (6.5) | <0.001 | 437 (18.4) | 23,465 (12.6) | <0.001 |
| Chest pain | 149 (14.7) | 12,643 (7.9) | <0.001 | 313 (13.2) | 15,462 (8.3) | <0.001 |
| Muscle/joint pain | 191 (18.9) | 15,903 (10.0) | <0.001 | 678 (28.6) | 32,555 (17.4) | <0.001 |

Data as n (%).

**S table 7.** Period prevalence during the pandemic (2020-2023) period of the relevant symptoms in men and women with a record of an acute SARS-CoV-2 infection who had not been hospitalized with a SARS-CoV-2 infection comparing the long COVID ≥ 90 days after group with the no long COVID ≥ 90 days after group.

|  | Men | | | Women | | |
| --- | --- | --- | --- | --- | --- | --- |
|  | Long COVID ≥ 90 days  n = 1,011 | No long COVID  ≥ 90 days  n = 159,293 | p-value | Long COVID  ≥ 90 days  n = 2,370 | No long COVID  ≥ 90 days  n = 186,895 | p-value |
| **Any of the symptoms** | 945 (93.5) | 76,828 (48.2) | <0.001 | 2,275 (96.0) | 119,191 (63.8) | <0.001 |
| **Cardiopulmonary** | 719 (71.1) | 41,608 (26.1) | <0.001 | 1,779 (75.1) | 66,532 (35.6) | <0.001 |
| Dyspnea | 401 (39.7) | 11,964 (7.5) | <0.001 | 1,059 (44.7) | 19,511 (10.4) | <0.001 |
| Cough | 539 (53.3) | 31,681 (19.9) | <0.001 | 1,248 (52.7) | 49,380 (26.4) | <0.001 |
| Palpitations | 135 (13.4) | 6,186 (3.9) | <0.001 | 558 (23.5) | 14,908 (8.0) | <0.001 |
| **CNS** | 709 (70.1) | 37,359 (23.5) | <0.001 | 1,799 (75.9) | 69,936 (37.4) | <0.001 |
| Fatigue | 611 (60.4) | 25,606 (16.1) | <0.001 | 1,571 (66.3) | 52,885 (28.3) | <0.001 |
| Cognitive dysfunction | 21 (2.1) | 2,108 (1.3) | 0.051 | 54 (2.3) | 2,768 (1.5) | 0.002 |
| Sleep disorder | 232 (22.9) | 15,133 (9.5) | <0.001 | 637 (26.9) | 26,899 (14.4) | <0.001 |
| Loss of smell/taste | 42 (4.2) | 858 (0.5) | <0.001 | 103 (4.3) | 1,352 (0.7) | <0.001 |
| **Pain** | 525 (51.9) | 36,928 (23.2) | <0.001 | 1,472 (62.1) | 62,901 (33.7) | <0.001 |
| Headache | 280 (27.7) | 15,195 (9.5) | <0.001 | 896 (37.8) | 32,925 (17.6) | <0.001 |
| Chest pain | 246 (24.3) | 15,719 (9.9) | <0.001 | 586 (24.7) | 19,848 (10.6) | <0.001 |
| Muscle/joint pain | 209 (20.7) | 13,956 (8.8) | <0.001 | 695 (29.3) | 28,384 (15.2) | <0.001 |

Data as n (%).

**S table 8.** Period prevalence during the pre-pandemic (2016-2019) period of the relevant symptoms in men and women with a record of an acute SARS-CoV-2 infection who had been hospitalized with a SARS-CoV-2 infection comparing the long COVID ≥ 90 days after group with the no long COVID ≥ 90 days after group.

|  | Men | | | Women | | |
| --- | --- | --- | --- | --- | --- | --- |
|  | Long COVID  n = 578 | No long COVID  n = 10,648 | p-value | Long COVID  n = 432 | No long COVID  n = 8,075 | p-value |
| **Any of the symptoms** | 362 (62.6) | 6,458 (60.6) | 0.365 | 335 (77.5) | 5,741 (71.1) | 0.005 |
| **Cardiopulmonary** | 212 (36.7) | 3,643 (34.2) | 0.242 | 202 (46.8) | 3,437 (42.6) | 0.095 |
| Dyspnea | 68 (11.8) | 1,645 (15.4) | 0.019 | 70 (16.2) | 1,647 (20.4) | 0.040 |
| Cough | 168 (29.1) | 2,519 (23.7) | 0.004 | 160 (37.0) | 2,254 (27.9) | <0.001 |
| Palpitations | 24 (4.2) | 410 (3.9) | 0.798 | 38 (8.8) | 702 (8.7) | 1.000 |
| **CNS** | 161 (27.9) | 3,255 (30.6) | 0.182 | 198 (45.8) | 3,322 (41.1) | 0.060 |
| Fatigue | 103 (17.8) | 1916 (18.0) | 0.960 | 147 (34.0) | 2,085 (25.8) | <0.001 |
| Cognitive dysfunction | 7 (1.2) | 434 (4.1) | 0.081 | S (1.2) | 324 (4.0) | 0.004 |
| Sleep disorder | 77 (13.3) | 1,522 (14.3) | 0.555 | 82 (19.0) | 1,716 (21.3) | 0.287 |
| Loss of smell/taste | 1 (0.2) | 15 (0.1) | 1.000 | 2 (0.5) | 15 (0.2) | 0.481 |
| **Pain** | 204 (35.3) | 3,242 (30.4) | 0.016 | 227 (52.5) | 3,260 (40.4) | <0.001 |
| Headache | 55 (9.5) | 791 (7.4) | 0.077 | 89 (20.6) | 1,045 (12.9) | <0.001 |
| Chest pain | 97 (16.8) | 1,627 (15.3) | 0.360 | 92 (21.3) | 1,334 (16.5) | 0.012 |
| Muscle/joint pain | 113 (19.6) | 1,676 (15.7) | 0.017 | 144 (33.3) | 1,971 (24.4) | <0.001 |

Data as n (%).

**S table 9.** Period prevalence during the pandemic (2020-2023) period of the relevant symptoms in men and women with a record of an acute SARS-CoV-2 infection who had been hospitalized with a SARS-CoV-2 infection comparing the long COVID ≥ 90 days after group with the no long COVID ≥ 90 days after group.

|  | Men | | | Women | | |
| --- | --- | --- | --- | --- | --- | --- |
|  | Long COVID  n = 578 | No long COVID  n = 10,648 | p-value | Long COVID  n = 432 | No long COVID  n = 8,075 | p-value |
| **Any of the symptoms** | 538 (93.1) | 8,340 (78.3) | <0.001 | 415 (96.1) | 6,638 (82.2) | <0.001 |
| **Cardiopulmonary** | 458 (79.2) | 5,590 (52.5) | <0.001 | 363 (84.0) | 4,506 (55.8) | <0.001 |
| Dyspnea | 381 (65.9) | 3,919 (36.8) | <0.001 | 291 (67.4) | 3,159 (39.1) | <0.001 |
| Cough | 274 (47.4) | 2,994 (28.1) | <0.001 | 253 (58.6) | 2,374 (29.4) | <0.001 |
| Palpitations | 62 (10.7) | 500 (4.7) | <0.001 | 92 (21.3) | 734 (9.1) | <0.001 |
| **CNS** | 355 (61.4) | 5,093 (47.8) | <0.001 | 313 (72.5) | 4,428 (54.8) | <0.001 |
| Fatigue | 275 (47.6) | 3,492 (32.8) | <0.001 | 261 (60.4) | 3,020 (37.4) | <0.001 |
| Cognitive dysfunction | 32 (5.5) | 1,039 (9.8) | 0.001 | 16 (3.7) | 778 (9.6) | <0.001 |
| Sleep disorder | 152 (26.3) | 2,042 (19.2) | <0.001 | 130 (30.1) | 2,051 (25.4) | 0.034 |
| Loss of smell/taste | 10 (1.7) | 43 (0.4) | <0.001 | 11 (2.5) | 35 (0.4) | <0.001 |
| **Pain** | 255 (44.1) | 3,205 (30.1) | <0.001 | 268 (62.0) | 3,054 (37.8) | <0.001 |
| Headache | 105 (18.2) | 945 (8.9) | <0.001 | 126 (29.2) | 1,188 (14.7) | <0.001 |
| Chest pain | 135 (23.4) | 1,858 (17.4) | <0.001 | 140 (32.4) | 1,423 (17.6) | <0.001 |
| Muscle/joint pain | 104 (18.0) | 1,194 (11.2) | <0.001 | 137 (31.7) | 1,400 (17.3) | <0.001 |

Data as n (%).

**S table 10.** Characteristics of the individuals who died before the end of the study period.

|  | n = 60,092 |
| --- | --- |
| Days between death date and start of study period, mean (SD) | 715 (413) |
| Age, mean (sd) | 79 (13) |
| Women, n (%) | 29,827 (49.6) |
| Long COVID, n (%) | 220 (0.4) |
| SARS-CoV-2 infection, n (%) | 15,358 (25.6) |
| Hospitalized with SARS-CoV-2 infection, n (%) | 5,418 (9.0) |
| Severe infection, n (%) | 921 (1.5) |

**S table 11.** Number of healthcare contacts overall during 2020-2023 in the hospitalized population.

|  | Men | | Women | |
| --- | --- | --- | --- | --- |
|  | Long COVID | No long COVID | Long COVID | No long COVID |
| Healthcare contacts | 107,302 | 1,064,854 | 97,026 | 913,524 |
| mean per patient | 117.8 (87.8) | 103.2 (91.2) | 146.3 (100.6) | 116.5 (96.7) |

Data as n, mean (sd)

**S table 12.** Adjusted OR from logistic regression models of association between occurrence of symptoms pre-pandemically and receiving a long COVID diagnosis in men and women in the non-hospitalized population. Univariate model and the multivariable models are used. The multivariable models include age group, obesity, asthma and anxiety in addition to the symptom of interest.

### Men

| **Symptom** | **Univariate model** | **Multivariable model** |
| --- | --- | --- |
| any | 2.39 (2.21–2.58) | 2.28 (2.10–2.48) |
| Cardiopulmonary | 2.10 (1.93–2.28) | 1.99 (1.82–2.17) |
| CNS | 2.07 (1.90–2.26) | 1.92 (1.75–2.11) |
| Pain | 2.10 (1.93–2.28) | 1.91 (1.76–2.08) |
| Dyspnea | 1.67 (1.44–1.94) | 1.58 (1.35–1.84) |
| Cough | 2.20 (2.01–2.40) | 2.07 (1.88–2.27) |
| Palpitations | 1.89 (1.58–2.25) | 1.61 (1.34–1.93) |
| Fatigue | 2.38 (2.16–2.63) | 2.20 (1.99–2.44) |
| Sleep disorder | 1.68 (1.48–1.91) | 1.39 (1.21–1.59) |
| Headache | 2.08 (1.83–2.36) | 1.81 (1.59–2.06) |
| Chest pain | 1.87 (1.66–2.10) | 1.69 (1.50–1.90) |
| Muscle/joint pain | 2.17 (1.96–2.40) | 1.96 (1.77–2.18) |

### Women

| **Symptom** | **Univariate model** | **Multivariable model** |
| --- | --- | --- |
| any | 2.48 (2.33–2.64) | 2.32 (2.18–2.48) |
| Cardiopulmonary | 1.89 (1.79–2.01) | 1.77 (1.67–1.88) |
| CNS | 2.03 (1.92–2.14) | 1.87 (1.76–1.98) |
| Pain | 2.11 (1.99–2.23) | 1.88 (1.77–1.99) |
| Dyspnea | 1.58 (1.44–1.73) | 1.51 (1.37–1.66) |
| Cough | 1.90 (1.79–2.02) | 1.73 (1.63–1.85) |
| Palpitations | 1.65 (1.50–1.81) | 1.49 (1.36–1.65) |
| Fatigue | 2.22 (2.09–2.35) | 1.95 (1.83–2.07) |
| Sleep disorder | 1.47 (1.36–1.59) | 1.32 (1.22–1.43) |
| Headache | 2.06 (1.92–2.22) | 1.72 (1.59–1.84) |
| Chest pain | 1.77 (1.63–1.92) | 1.66 (1.53–1.81) |
| Muscle/joint pain | 1.99 (1.87–2.12) | 1.74 (1.63–1.85) |

**S table 13.** Adjusted OR from logistic regression models of association between number of symptoms pre-pandemically and receiving a long COVID diagnosis in men and women in the non-hospitalized population. Number of symptoms was categorized into zero, one, two, three and more than three symptoms. Having zero symptoms was used as reference. Univariate model and the multivariable models are used. The multivariable models include age group, obesity, asthma and anxiety in addition to the number of symptoms.

### Men

| **Number of symptoms** | **Univariate model** | **Multivariable model** |
| --- | --- | --- |
| 1 | 1.92 (1.74–2.11) | 1.88 (1.71–2.07) |
| 2 | 2.75 (2.46–3.08) | 2.71 (2.41–3.05) |
| 3 | 3.27 (2.81–3.80) | 3.21 (2.74–3.75) |
| >3 | 4.52 (3.78–5.41) | 4.34 (3.60–5.23) |

### Women

| **Number of symptoms** | **Univariate model** | **Multivariable model** |
| --- | --- | --- |
| 1 | 1.91 (1.77–2.05) | 1.87 (1.74–2.02) |
| 2 | 2.55 (2.35–2.76) | 2.48 (2.29–2.70) |
| 3 | 3.28 (2.99–3.61) | 3.16 (2.87–3.48) |
| >3 | 4.26 (3.86–4.70) | 4.04 (3.63–4.49) |

**S table 14.** Odds ratios (OR) and 95% confidence intervals (CI) for baseline comorbidities using logistic regression with no Long COVID as reference. The unadjusted odds ratio is derived from a univariate model. The adjusted model is a bivariate model with age added as a dependent variable.

|  | Men | | Women | |
| --- | --- | --- | --- | --- |
|  | Unadjusted | Adjusted | Unadjusted | Adjusted |
| Type_1 Diabetes | 1.01 (0.74 – 1.38) | 1.06 (0.77 – 1.45) | 0.77 (0.57 – 1.04) | 0.87 (0.64 – 1.17) |
| Type_2 Diabetes | 0.96 (0.82 – 1.13) | 1.24 (1.05 – 1.46) | 0.65 ( 0.55 – 0.76) | 1.00 ( 0.85 – 1.17) |
| Obesity | 1.64 (1.43 – 1.88) | 1.78 (1.55 – 2.04) | 1.37 (1.25 – 1.50) | 1.46 (1.33 – 1.60) |
| Dyslipidemia | 0.97 (0.87 – 1.09) | 1.30 (1.15 – 1.47) | 0.62 (0.56 – 0.69) | 1.06 (0.95 – 1.18) |
| Hypertension | 0.95 (0.86 – 1.05) | 1.29 (1.15 – 1.44) | 0.68 (0.63 – 0.74) | 1.27 ( 1.17 – 1.39) |
| Heart failure | 0.64 (0.47 – 0.88) | 1.13 (0.81 – 1.56) | 0.33 (0.23 – 0.47) | 0.92 (0.65 – 1.32) |
| Atrial fibrillation | 0.68 (0.55 – 0.86) | 1.14 (0.89 – 1.44) | 0.38 (0.30 – 0.50) | 0.97 ( 0.74 – 1.26) |
| Coronary heart disease | 0.78 (0.64 – 0.94) | 1.23 (1.00 – 1.50) | 0.50 (0.40 – 0.63) | 1.12 (0.89 – 1.41) |
| Stroke | 0.60 (0.40 – 0.90) | 0.90 (0.60 – 1.35) | 0.54 (0.39 – 0.76) | 1.11 (0.79 – 1.56) |
| Peripheral vascular disease | 0.57 (0.28 – 1.14) | 0.96 (0.48 – 1.93) | 0.32 (0.16 – 0.64) | 0.70 (0.35 – 1.41) |
| Pulmonary embolism | 1.27 (0.79 – 2.05) | 1.63 (1.01 – 2.63) | 0.71 (0.44 – 1.12) | 1.10 (0.69 – 1.75) |
| Deep vein thrombosis | 1.22 (0.88 – 1.71) | 1.46 (1.05 – 2.05) | 0.90 (0.70 – 1.14) | 1.20 (0.94 – 1.53) |
| COPD | 0.72 (0.52 – 1.00) | 1.06 (0.76 – 1.47) | 0.60 (0.48 – 0.75) | 1.01 (0.80 – 1.27) |
| Asthma | 1.64 (1.43 – 1.88) | 1.65 (1.44 – 1.89) | 1.82 (1.68 – 1.97) | 1.90 (1.76 – 2.06) |
| Chronic kidney disease | 0.65 (0.46 – 0.91) | 1.10 (0.77 – 1.57) | 0.28 ( 0.18 – 0.43) | 0.67 ( 0.44 – 1.04) |
| Dementia | 0.26 (0.11 – 0.57) | 0.58 (0.26 – 1.30) | 0.22 (0.13 – 0.36) | 0.72 ( 0.43 – 1.21) |
| Depression | 1.89 (1.69 – 2.10) | 1.89 (1.69 – 2.10) | 1.72 (1.61 – 1.84) | 1.76 (1.65 – 1.88) |
| Anxiety | 1.86 (1.68 – 2.06) | 1.86 (1.68 – 2.06) | 1.85 (1.74 – 1.96) | 1.85 (1.74 – 1.97) |

Data as OR (95% CI)


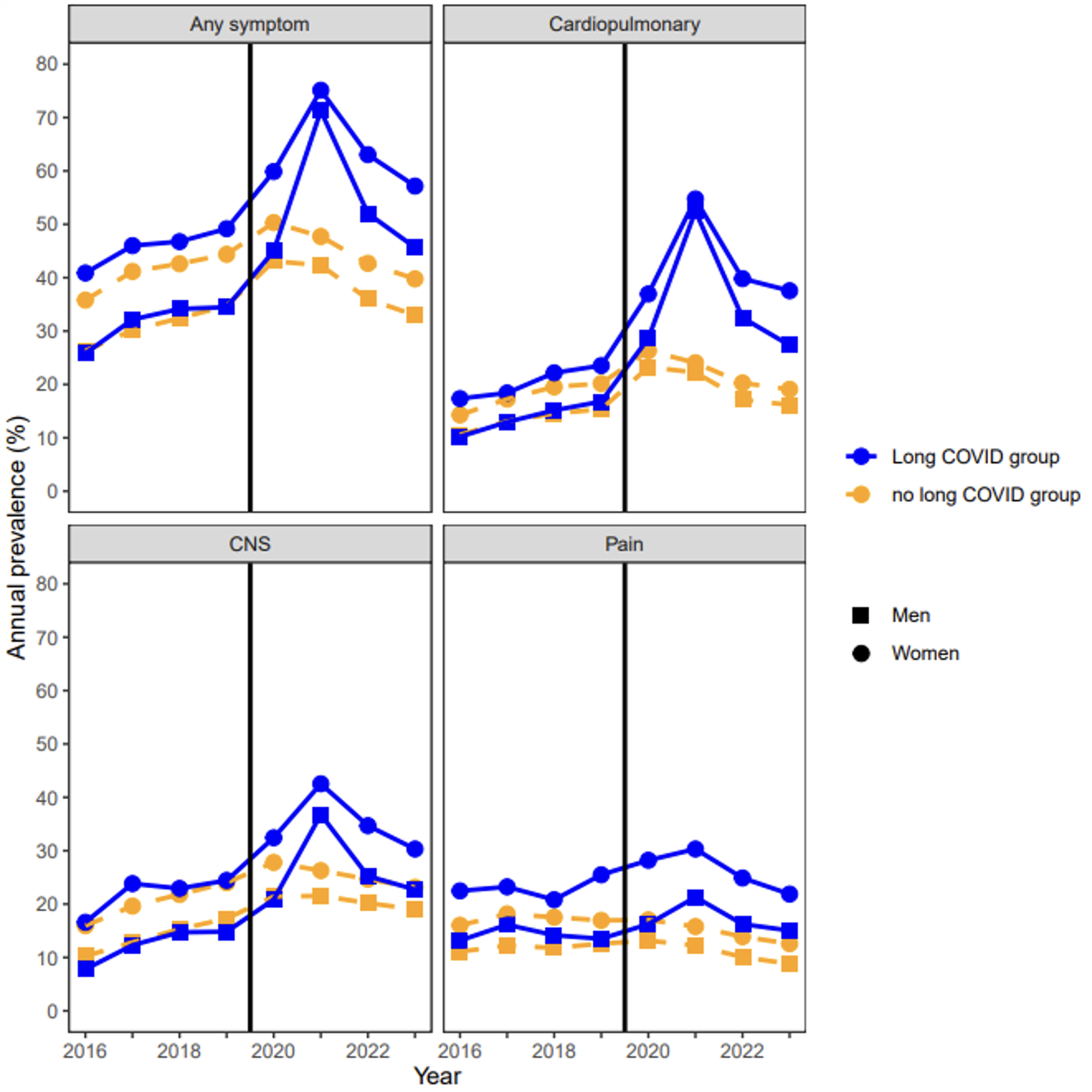


**S figure 1.** Annual prevalence of any (one or more) of the important symptoms overall and in each symptom category in men (squares) and women (circles) hospitalized with a SARS-CoV-2 infection, comparing the long COVID group (blue, solid lines) vs the no long COVID group (orange, dashed lines). The black vertical line marks the transition from the pre-pandemic period into the pandemic period.


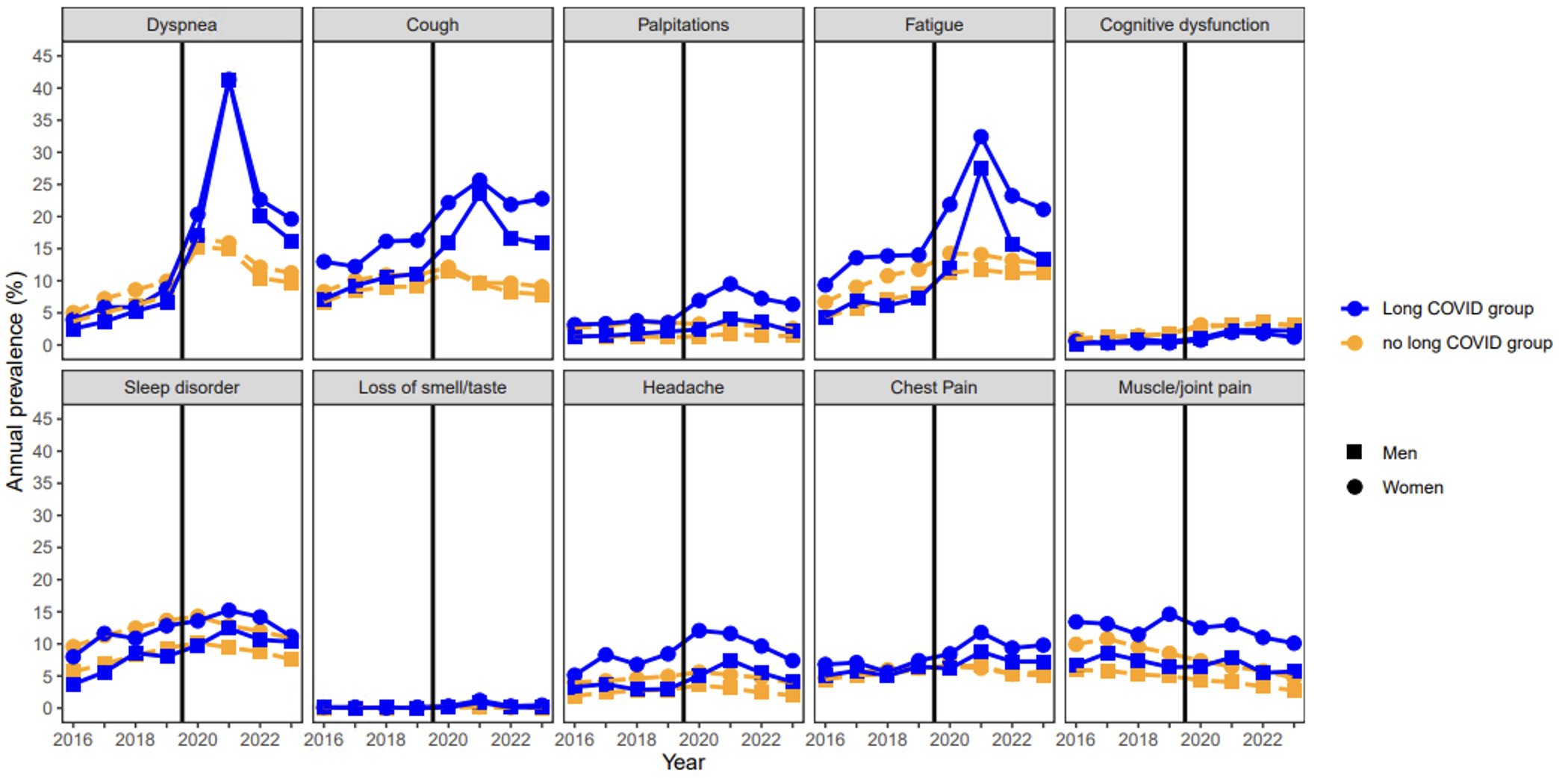


**S Figure 2.** Annual prevalence of the important symptoms in men (squares) and women (circles) hospitalized with a SARS-CoV-2 infection, comparing the long COVID group (blue, solid lines) vs the no long COVID group (orange, dashed lines). The black vertical line marks the transition from the pre-pandemic period into the pandemic period.


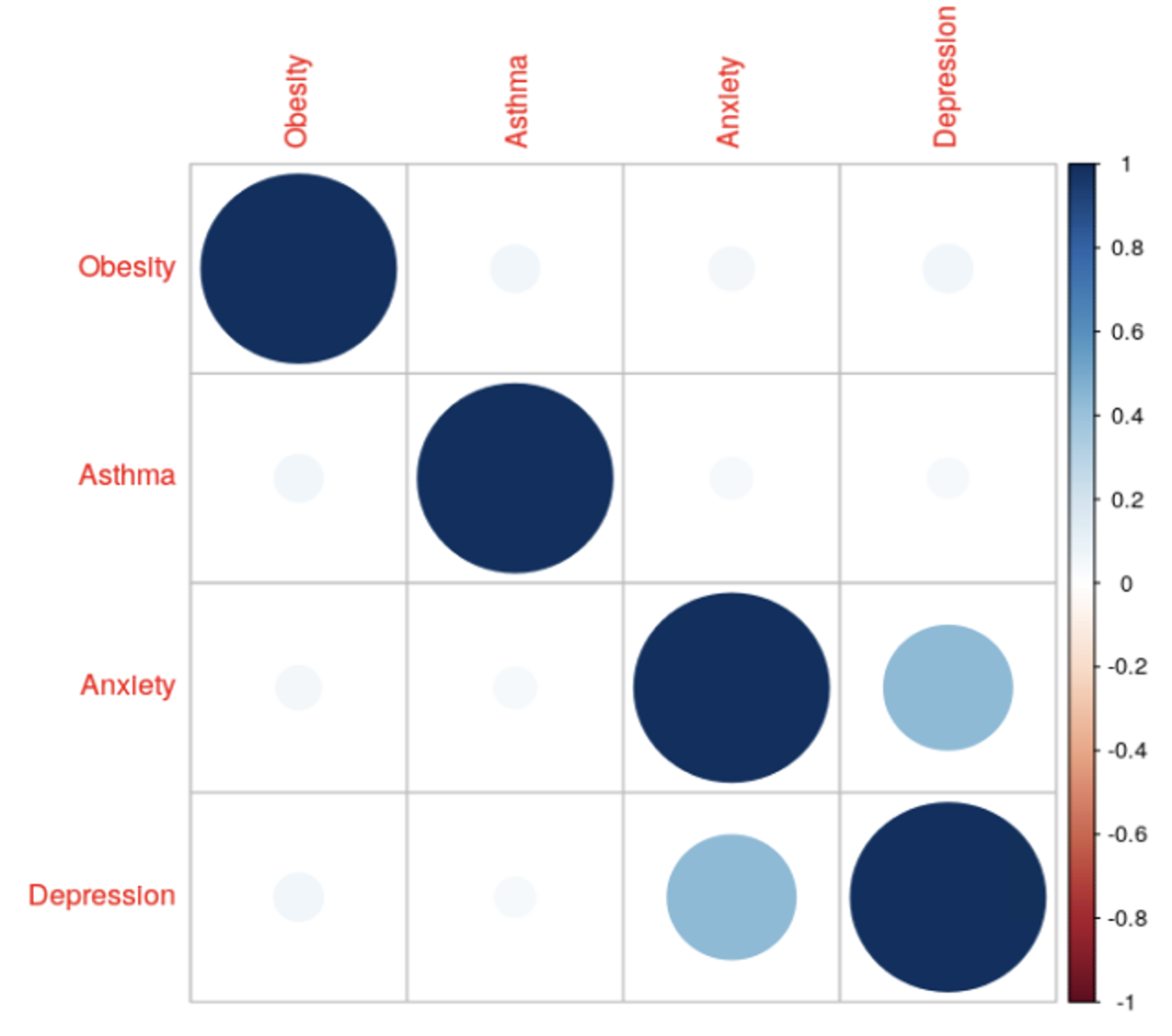


**S Figure 3.** Correlation matrix of the baseline comorbidities with a count of 100 or more individuals in the long COVID group that had statistically significant difference in prevalence compared with the no long COVID group in both men and women. The plot looks identical for men and women. Size and color of the circles corresponds to the magnitude of the correlation (larger and darker color represents a stronger correlation).

**References**

1. Soriano JB, Murthy S, Marshall JC, Relan P, Diaz JV, Condition WHOCCDWGoP-C-. A clinical case definition of post-COVID-19 condition by a Delphi consensus. *Lancet Infect Dis* 2022; **22**(4): e102-e7.
